# Hypercapnia Induces Mitochondrial Adaptations and Alters Glutamine Metabolism to Drive a Distinct Metabolic Phenotype in Monocytes

**DOI:** 10.1111/imcb.70162

**Published:** 2026-09-16

**Authors:** Ben Reddan, David E. Phelan, Xiaofei Yin, Lorraine Brennan, Martina Wallace, Eoin P. Cummins

**Affiliations:** ^1^ School of Medicine University College Dublin Dublin Ireland; ^2^ Conway Institute of Biomolecular and Biomedical Science University College Dublin Dublin Ireland; ^3^ School of Agriculture and Food Science University College Dublin Dublin Ireland

## Abstract

Carbon dioxide (CO_2_) is an ancient and ubiquitous physiological gas generated during aerobic respiration. Historically viewed as a simple metabolic waste product, CO_2_ has received far less research attention than oxygen (O_2_), the primary substrate of aerobic respiration. However, emerging evidence has revealed important roles for CO_2_ in immunometabolism, immunology, muscle physiology, and clinical medicine. While circulating pCO_2_ levels are tightly regulated, patients with lung diseases such as chronic obstructive pulmonary disease (COPD) frequently develop hypercapnia, pCO_2_ > 45 mmHg. Hypercapnia is associated with significantly increased mortality, higher risk of ICU admission, and a global prevalence estimated at 13–15 million patients. Its broader clinical consequences remain poorly understood and are inadequately integrated into current therapeutic paradigms. Here, we examined the impact of hypercapnia on the metabolic profile of monocytes. We demonstrate that 24 h of buffered hypercapnia induces a marked reduction in mitochondrial mass. This is accompanied by dysregulation of mitochondrial membrane potential and key bioenergetic substrates (NADH/NAD^+^ and ATP content). We further show that hypercapnia alters the abundance of metabolites and proteins associated with mitochondrial metabolism, with effects spanning glucose, glutamine, and lipid metabolism. Thus, we provide direct mechanistic evidence that hypercapnia directly alters the glutamine–glutamate–proline synthesis axis. Collectively, these findings establish the foundation for a discrete hypercapnic metabolic phenotype, that is, in several respects, distinct from the metabolic adaptations observed in hypoxia. We propose that hypercapnia triggers a cascade of metabolic adaptations with tissue‐dependent consequences on cellular effector functions.

## Graphical abstract

Elevated CO_2_ (hypercapnia) is a feature of serious lung diseases, for example COPD and contributes to poor patient prognosis through modulation of immune cell function. Here we employ state‐of‐the art metabolomic and isotope tracing approaches to reveal CO_2_‐dependent mitochondrial alterations linked to suppressed ATP, proline and glutamate levels in monocytes. This distinct metabolic phenotype gives insight into the metabolic dysfunction associated with patients experiencing hypercapnia.

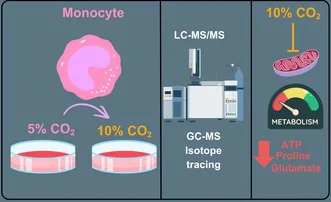

## 1 Introduction

Molecular oxygen (O_2_) serves as the primary gaseous substrate for cellular metabolism, while carbon dioxide (CO_2_) is its principal gaseous by‐product. Gas exchange between these molecules occurs via passive diffusion in the lungs, where O_2_ and CO_2_ transfer across the alveolar–capillary interface. The efficiency of pulmonary gas exchange is dictated by both the rate of blood perfusion through the capillaries and the diffusion capacity of the alveolar membrane [1]. Once across the alveolar barrier, hemoglobin (Hb) in erythrocytes facilitates systemic O_2_ delivery and CO_2_ clearance [2]. Disturbances in O_2_ or CO_2_ homeostasis present serious risks to cellular function and organismal viability. Pulmonary pathologies such as chronic obstructive pulmonary disease (COPD) and acute respiratory distress syndrome (ARDS) can impair lung architecture and function, disturbing the delicate balance of O_2_/CO_2_ exchange. Under normal physiological conditions, arterial pCO_2_ (normocapnia) typically ranges between 35 and 45 mmHg; however, CO_2_ levels within tissues can vary considerably depending on local metabolic activity and diffusion capacity. The pathological state of hypercapnia is defined as an arterial pCO_2_ > 45 mmHg, which arises from respiratory insufficiency or altered metabolic output [3]. A hypercapnic microenvironment typically develops due to restricted CO_2_ elimination and/or enhanced CO_2_ production and has been reported in respiratory disease patients (i.e., COPD) as well as solid tumors, where tissue pCO_2_ levels can range between 59 and 84 mmHg [4].

The most common treatments for hypercapnia include both bi‐level positive airway pressure (BiPAP) [5] and extracorporeal CO_2_ removal (ECCO_2_R) [6]. Epidemiological data underscore the significant burden of hypercapnia, whereby 29.7% of patients with severe COPD had baseline hypercapnia (PaCO_2_ ≥ 45 mmHg) [7]. Among GOLD stage III–IV COPD patients, 25% had PaCO_2_ ≥ 45 mmHg, and 9% had PaCO_2_ ≥ 50 mmHg [8]. Interestingly, a 12‐month cross‐sectional study investigating hypercapnic respiratory failure, including acute and chronic forms in individuals aged 15 years and older, reported an average adjusted annual period prevalence of 163 cases per 100 000 population, highlighting the substantial prevalence of hypercapnia and its association with severe respiratory dysfunction [9]. Clinically, hypercapnia represents a significant risk factor in patients with pulmonary disorders and is associated with an increased in‐hospital mortality of up to 17% and a 1‐year mortality rate of up to 32% [10]. This highlights the importance of developing a deeper understanding of the role this pulmonary condition plays in driving downstream outcomes for patients.

A growing body of evidence demonstrates that elevated CO_2_ exerts broad effects on cellular metabolism, extending beyond any single individual pathway. Hypercapnia reprograms both amino acid and lipid metabolism and further dysregulates mitochondrial dynamics, as shown by transcriptional, morphological, metabolic, and functional analyses. For example, exposure to 15% CO_2_ suppresses IDH2 protein expression via miR‐183, impairing proliferation in fibroblasts and epithelial cancer cells—an effect rescued by α‐ketoglutarate supplementation [11]. Additionally, short‐term exposure (4 h) to 10% CO_2_ under pH‐buffered conditions reduces *mRNA* levels of the glycolytic enzyme ALDOA and proline synthesis enzyme PYCR1 in THP‐1 monocytes, while further upregulating the *mRNA* levels of mitochondrial genes associated with respiratory complexes I and IV [12]. Moreover, prolonged hypercapnia significantly alters gene expression associated with lipid metabolism pathways in both primary human bronchial epithelial cells (24 h, 20% CO_2_) and THP‐1 monocytes (24 h, 10% CO_2_). Beyond immune and epithelial cells, hypercapnia also contributes to muscle dysfunction—a recognized feature of COPD—by accelerating muscle atrophy [13] and upregulating fatty acid oxidation in murine muscle cells [14]. By reshaping key metabolic pathways, elevated CO_2_ not only perturbs metabolic homeostasis but also sets the stage for broader effector function consequences, highlighting a critical intersection between metabolism and immune regulation that will be explored in the following section.

While the literature is broadly consistent regarding metabolic changes associated with hypercapnia, the field remains fragmented by differences in CO_2_ exposure concentrations (5%–20% CO_2_), durations of exposure (ranging from minutes to days), and experimental models (buffered hypercapnia versus hypercapnic acidosis). Here, we sought to address this through a unified and systematic approach, providing an in‐depth characterization of CO_2_‐dependent metabolic changes within a homogeneous model. Expanding on our previous work, which identified early transcriptional changes linked to mitochondrial DNA, amino acid and lipid metabolism, and altered metabolite abundance [12], we performed a deeper investigation of hypercapnic responses at the protein level, alongside a comprehensive assessment of mitochondrial biomolecular composition, functional status and ^13^C‐nutrient incorporation after 24 h. Our aim was to define a thoroughly characterized hypercapnic metabolic phenotype, analogous to that described in hypoxia. We anticipate that our experimental approach will provide novel insights into the complex metabolic signaling networks that may lead to detrimental effector function outcomes. Our “omic” data will also be a valuable resource to the research community for the pursuit of future metabolism‐focused studies.

To isolate CO_2_‐specific effects from pH‐related changes, all experiments were performed under tightly controlled pH‐buffered conditions. Monocytes were selected as the primary model due to their central role in pulmonary immunity, accessibility in the circulation as a potential surrogate source for hypercapnia diagnostics, and because they are among the few immune cell types previously studied under hypercapnic conditions [12, 15, 16, 17]. In vivo, physiological CO_2_ and O_2_ gradients exist across tissues, with deeper regions often reflecting a hypoxic–hypercapnic *milieu*. Given their migratory nature, monocytes must adapt to dynamic gaseous niches, making them a relevant model to investigate metabolic responses to elevated CO_2_. Although this study does not directly model COPD, hypercapnia occurs across multiple pulmonary disorders. We therefore focused on defining CO_2_‐specific responses in monocytes while avoiding confounding disease‐specific factors associated with conditions such as COPD, ARDS, and neuromuscular disease.

## 2 Materials and Methods

### 2.1 THP‐1 Monocytes

THP‐1 monocytes were maintained at a density of 2 × 10^5^–1 × 10^6^ cells/ml and cultured twice per week, with passage number continuously tracked. All experiments were carried out below P30 to prevent unwanted effects due to aging. All procedures and treatments prior to cell lysis were performed in a Class II biological safety cabinet or a CO_2_ chamber, as described below. For cell culture, cells were counted and media containing the appropriate number of cells were transferred to a T75 tissue culture flask containing fresh media to a total of 20–30 mL. THP‐1 cells were maintained in RPMI‐1640 medium (25 mM Glucose, 4 mM Glutamine) (61870036, Gibco), supplemented with 10% fetal bovine serum (FBS) (10270‐106, Gibco) and 1% Penicillin–Streptomycin (PenStrep) (15070‐063, Gibco). Cells were maintained in incubators (Thermo Scientific) at 37°C in 21% O_2_ and 5% CO_2_.

### 2.2 Nonyl Acridine Orange Staining

Acridine orange 10‐nonyl bromide (A1372, Merck) was applied to assess mitochondrial cardiolipin content [18]. Cardiolipin is a phospholipid specifically present on the mitochondrial membrane, which strongly correlates with mitochondrial mass. NAO emission can be detected at 519 nm. In brief, THP‐1 monocyte cells were seeded on flat‐bottomed six‐well plates in 2 mL phenol‐free RPMI‐1640 GlutaMAX medium. Subsequently, a final concentration of 500 nM of NAO/PBS was prepared and added to each well in a six‐well plate at a ratio of 1:2 (NAO/PBS: Media). Cells were incubated at 37°C and 5% CO_2_ for 30 min, protected from light. Cells were then washed to remove excess dye with 100 μL of PBS and prepared for flow cytometry by resuspending in 100 μL PBS. Analysis was conducted on a CytoFLEX LX flow cytometer (Beckman Coulter Life Sciences). Background was removed by subtracting the negative control (cells without NAO dye) value from all samples (Figure S1). Mitochondrial dye staining was performed in parallel using the same biological replicates but on separate duplicate samples rather than by co‐staining. This approach minimized potential dye–dye interactions, spectral overlap, and perturbation of mitochondrial physiology, thereby ensuring accurate and independent assessment of each mitochondrial parameter under identical experimental conditions.

### 2.3 BioTracker 405 Staining

BioTracker 405 (SCT135, Sigma‐Aldrich) is a fluorogenic dye used for staining mitochondria. The dye is membrane‐permeable and accumulates within mitochondria becoming brightly fluorescent. BioTracker 405 is specifically designed for assessing mitochondrial membrane potential (ΔΨm), a critical indicator of mitochondrial function. The chemical structure of BioTracker 405 is not disclosed by the manufacturer. BioTracker405 emission can be detected at 440 nm. In brief, THP‐1 monocyte cells were seeded in respective 35 mm plates in 2 mL of phenol‐free RPMI‐1640 GlutaMAX media and incubated for 24 h. Subsequently, a final concentration of 100 nM (flow cytometry) or 400 nM (microscopy) of BioTracker405 was spiked into the media. Cells were incubated at 37°C and 5% CO_2_ for 30 min protected from light. Cells were then prepared and analyzed by flow cytometry (Figure S1). Mitochondrial dye staining was performed in parallel using the same biological replicates but on separate duplicate samples rather than by co‐staining. This approach minimized potential dye–dye interactions, spectral overlap, and perturbation of mitochondrial physiology, thereby ensuring accurate and independent assessment of each mitochondrial parameter under identical experimental conditions.

### 2.4 ATP Assay Quantification

The CellTiter‐Glo assay quantifies intracellular ATP levels. Although it is frequently used as an indirect measure of cell viability—since ATP production typically correlates with the number of viable cells—the assay primarily measures ATP abundance. Briefly, approximately 1 × 10^6^ THP‐1 cells were exposed to 5% CO_2_ or 10% CO_2_ in appropriately buffered DMEM for 24 h. As per the manufacturer's protocol, following exposure, 100 μL of the cell suspension was transferred to a 96‐well plate, and CellTiter‐Glo reagent (Promega, G9241) was added at a 1:1 media‐to‐reagent ratio. Plates were incubated for 2 min at room temperature on an orbital shaker, protected from light, to facilitate cell lysis. Samples were then incubated for a further 10 min at room temperature, protected from light. Luminescence was recorded using a CLARIOstar plate reader (BMG Labtech). Background signal was corrected by subtracting readings from negative control wells containing a cell‐free medium with CellTiter‐Glo reagent. All measurements were performed in triplicate.

### 2.5 NADH/NAD ^+^ Assay Quantification

Intracellular NAD^+^ and NADH levels (total NAD^+^ and NADH) were measured using the NAD/NADH Assay Kit (Colorimetric) (Abcam, ab65348) according to the manufacturer's instructions with minor modifications. Cells were seeded at an initial density optimized for the assay. Briefly, the required number of cells (2 × 10^5^ cells) was seeded. Cells were washed once with ice‐cold PBS, pelleted by centrifugation at 100 × g for 5 min at 4°C, and the supernatant was discarded. Cell pellets were lysed in 400 μL of Extraction Buffer II using two freeze–thaw cycles (20 min on dry ice followed by 10 min at room temperature). Lysates were vortexed for 10 s and centrifuged at maximum speed for 5 min at 4°C in a prechilled microcentrifuge to remove insoluble debris. The supernatant containing extracted NAD^+^/NADH was transferred to a fresh tube and kept on ice. To remove enzymes that rapidly consume NADH, samples were deproteinized by filtration through a 10 kDa spin column. The sample was loaded onto the spin column and centrifuged at 10 000 × g for 10 min at 4°C. The filtrate was collected and kept on ice. Samples were tested neat or at a 1:5 dilution in Extraction Buffer II, depending on the expected concentration range. Background signal was subtracted using negative control wells containing cell‐free medium mixed with the assay reagents. All samples and standards were measured in triplicate. Absorbance, indicative of the total combined NADH/NAD^+^ abundance, was recorded at 450 nm using a CLARIOstar microplate reader (BMG Labtech).

### 2.6 PrestoBlue Assay Quantification

Cell metabolic activity was assessed using the PrestoBlue Cell Viability Reagent (Thermo Fisher Scientific, A13261) according to the manufacturer's instructions with minor modifications. The PrestoBlue assay is a cell metabolic activity assay that is commonly used as an indirect measure of cell viability, proliferation, and cytotoxicity. It does not directly count living cells. This reduction is primarily driven by reductase activity. Cells were seeded at an assay‐optimized density and exposed to the indicated experimental conditions. Following treatment, PrestoBlue reagent was added at a ratio of 1:10 directly to the culture medium, and cells were incubated at 37°C for 10 min to allow reduction of the resazurin‐based reagent to fluorescent resorufin by metabolically active cells. Fluorescence intensity, proportional to cellular metabolic activity, was measured using a CLARIOstar microplate reader (BMG Labtech) with excitation and emission wavelengths of 560 nm and 590 nm, respectively. Background fluorescence was corrected using cell‐free medium containing PrestoBlue reagent only. Values were normalized to the appropriate vehicle control and expressed as relative metabolic activity or fold change where indicated. All samples and standards were measured in triplicate.

### 2.7 Western Blot Analysis

Whole‐cell protein lysates were prepared using whole‐cell lysis buffer (150 mM NaCl, 25 mM Tris pH 8, 1 mM ethylenediaminetetraacetic acid, 1% Triton X‐100). Cytosolic protein lysates were prepared using a hypotonic lysis buffer method. Cells were first incubated on ice for 10 min in cytosolic buffer (10 mM HEPES, pH 8; 1.5 mM MgCl_2_; 10 mM KCl; 200 mM sucrose; 0.5 mM DTT; 0.25% NP‐40 (IPEGAL); protease inhibitor cocktail (PIC) 1:100), then scraped and centrifuged. The resulting pellet was resuspended in nuclear lysis buffer (20 mM HEPES, pH 8; 420 mM NaCl; 0.2 mM EDTA; 1.5 mM MgCl_2_; 0.5 mM DTT; 25% glycerol; PIC 1:100) and incubated on ice for 30 min, followed by centrifugation. The supernatant, representing the nuclear protein fraction, was collected. All lysis buffers were supplemented with protease inhibitor cocktail (P2714, Merck). Mitochondrial lysates were prepared using a mitochondrial isolation kit (89874, Thermo Scientific) according to the manufacturer's instructions, with isolated mitochondria lysed in 2% CHAPS (Merck) in TrisBuffer Saline (TBS).

Lysates were quantified using the DC Protein Assay kit (Bio‐Rad, Hercules, CA, USA) before sodium dodecyl sulfate–polyacrylamide gel electrophoresis on the Bio‐Rad mini‐protean system. Wet transfer was performed onto nitrocellulose membranes and reversibly stained with Revert 700 total protein stain (Li‐COR, Lincoln, NE, USA). Membranes were washed in Revert 700 wash solution and imaged at 700 nm (Li‐COR), then destained in Revert 700 destaining solution (Li‐COR) prior to immunoblotting. Membranes were blocked in 5% milk in TBST for 1 h followed by overnight incubation in primary antibody (1:1000) at 4°C (PYCR1, Cell Signaling, 2535S; Glutaminase‐1, Cell Signaling, 56750T; TFAM, Cell Signaling, CST‐8076; Phospho‐Acetyl‐CoA (Ser79), Cell Signaling, 3661S; VDAC 1/2, ProteinTech, 10866‐1‐AP). Fluorescent secondary goat anti‐mouse or anti‐rabbit antibody (IgG (H + L) Goat anti‐Rabbit, DyLight 800 4X PEG: 10733944, Fisher Scientific, IgG (H + L) Cross‐Adsorbed Goat anti‐Mouse, DyLight 680: 10797775, Fisher Scientific) incubations were performed at a 1:2000 dilution and imaged at 680 nm or 800 nm. Membranes were imaged using a LiCor Odyssey CLx imager and analyzed using Empiria Studio image analysis software (LiCor, version 2.3.0.154). Nuclear fractions were immunoblotted for the protein of interest and Lamin A/C, with protein expression levels normalized to Lamin A/C as a loading control for nuclear protein content.

### 2.8 Cell Counting and Viability Assessments

A 10 μL aliquot of cells was drawn from each well and mixed 1:1 with 10 μL of trypan blue (15250‐061, Gibco) solution. The mixture was thoroughly mixed, and 10 μL was loaded onto a hemocytometer. Cells were counted in three fields, and the average count was calculated. To account for the 1:1 dilution with trypan blue, total cell numbers were multiplied by two. Viable cells were identified as clear (unstained) due to intact membranes, whereas nonviable cells appeared dark blue, indicating loss of membrane integrity.

### 2.9 Carbon Dioxide Exposures

All CO_2_ exposures were carried out in humidified environmental chambers (Coy Laboratories) maintained at 37°C. For atmospheric experiments, CO_2_ concentrations were set at 5% (physiological conc.) or 10% (pathophysiological conc.). Experiments utilized atmosphere‐specific buffered, phenol red‐free DMEM (D1152, Sigma–Aldrich) (4500 mg/L glucose, l‐glutamine, and 25 mM HEPES, 10% FBS Gibco #10270‐106, 1% Penicillin–Streptomycin) (PenStrep) (15070‐063, Gibco) supplemented with: 1.21 g NaHCO_3_/500 mL, 0.78 g NaCl/500 mL 5% CO_2_ media, or 2.21 g NaHCO_3_/500 mL, 0 g NaCl/500 mL 10% CO_2_ media (Table S1). Media was preequilibrated to the different CO_2_ environments in advance of experiments to ensure medium CO_2_/NaHCO_3_^−^ equilibrium prior to the initiation of experiments (Table S1). This model previously demonstrated that extracellular and intracellular pH remains stable in THP‐1 cells cultured under these pH‐buffered conditions [12, 16]. For all *in cellula* experiments, cells were seeded at the density indicated in the respective protocols below.

### 2.10 Cell Culture Protocols for Isotopic Labeling Experiments

Cells were initially cultured in DMEM‐5030 (D5050, Sigma–Aldrich) supplemented with 12.5 mM glucose, 2 mM glutamine, 10% FCS, and 1% penicillin/streptomycin. For experiments involving isotopic tracing, U‐^13^C‐glucose (389374‐1G, Sigma–Aldrich) was used in place of standard glucose while glutamine (25030‐024, Gibco) remained unlabeled, and conversely, U‐^13^C‐glutamine (605166‐100 mg, Sigma–Aldrich) was used with unlabeled glucose (G8270, Sigma–Aldrich). Media formulations were adjusted with the appropriate volumes of NaHCO_3_ and NaCl to accommodate both 5% and 10% CO_2_ exposures. Media were prebuffered several hours before the experiment to allow pH equilibration.

For the exposure experiments, 1 × 10^6^ cells were resuspended in 5% or 10% CO_2_^13^C‐labeled media, and incubated for 24 h. For experiments involving Roxadustat, at hour 4, Roxadustat (200 μM) (7486, Tocris) was added for the remaining 20 h of exposure. Following the 24‐h treatment, cells were collected by centrifugation and returned to the incubator without exposure to the standard 0.04% CO_2_ environment. Cell pellets were washed with 0.9% saline, transferred to 1.5 mL Eppendorf Safe‐Lock tubes (0030120086, Eppendorf), flash‐frozen in liquid nitrogen, and stored at −80°C.

For sample processing, frozen pellets were thawed on ice. To each pellet, 230 μL methanol, 20 μL methanol containing C16 d31 (1 nmol), 100 μL water with norvaline (1 μg), and 250 μL chloroform were added. Samples were vortexed for 5 min at 4°C and centrifuged at 13 000 × g for 5 min at 4°C. The resulting phases were separated into three layers: polar (upper), lipid (lower), and intermediate. Each phase was transferred to separate Eppendorf Safe‐Lock tubes (0030120086, Eppendorf) and dried under a nitrogen stream for 20–30 min.

### 2.11 GC–MS Analysis of ^13^C‐Glucose and ^13^C‐Glutamine Enrichment

Dried polar metabolites were derivatized in 2% (w/v) methoxyamine hydrochloride (Thermo Scientific) in pyridine and incubated at 37°C for 60 min. Samples were then silylated with *N*‐tert‐butyldimethylsilyl‐*N*‐methyltrifluoroacetamide (MTBSTFA) with 1% tert‐butyldimethylchlorosilane (tBDMCS) (Regis Technologies) at 45°C for 30 min. Polar derivatives were analyzed by GC–MS using a DB‐35MS UI column (30 m × 0.25 mm i.d. × 0.25 μm, Agilent J&W Scientific) installed in an Agilent 8890B gas chromatograph (GC) interfaced with an Agilent 5977 GC/MSD mass spectrometer (MS) with an XTR ion source. Inlet parameters: 1 μL was injected at 270°C with a purge flow to the split vent of 100 mL/min at 0.75 min. These were analyzed using the following temperature program: 100°C initially for 2 min, increase by 5°C/min to 215°C, increase by 10°C/min to 320°C, then hold for 4 min. The percentage isotopologue distribution of each polar metabolite was determined and corrected for natural abundance in MATLAB using algorithms adapted from Fernandez et al. [19]. Mole percent enrichment (MPE) was calculated via the following equation:

$$\frac{\sum_{i=1}^{n}M_{i}\cdot i}{n}$$

GC–MS summary data are included as Supplemental sheets 1–4 in Appendix S1.

### 2.12 LC–MS/MS Analysis of Lipid and Amino Acid Abundance

shNT THP‐1 monocytes (~750 k cells mL^−1^) were incubated at 37°C with either 5% or 10% CO_2_‐buffered DMEM (high glucose; Merck D1152 with 10% fetal bovine serum and 1% penicillin–streptomycin) for 24 h, with or without LPS (Invivogen, San Diego, CA, USA) (2.5 μg mL^−1^) for the final 22 h. The cell suspension was transferred to a 15 mL tube, and any attached cells were collected from the plate with a cell scraper and cold PBS. Cells were pelleted at 1000 g for 5 min and washed twice with ice‐cold PBS. The final cell pellet was snap‐frozen in liquid nitrogen before extraction. Metabolite extractions were performed to measure a broad range of metabolites from cell pellets (10 technical replicates per condition). In brief, metabolites were extracted with ethanol/phosphate buffer (85% v/v). The metabolites were measured using LC–MS/MS analysis and flow injection analysis–MS/MS analysis based on the p180biocrates assay as per the manufacture's instructions.

The data were acquired on an SCIEX QTRAP6500plus mass spectrometer coupled to SCIEX ExionLC Series UHPLC capability as previously described [20]. Liquid chromatography was performed using a custom ultra‐high‐performance liquid chromatography column with acetonitrile and water with 0.2% formic acid as the mobile phase. Amino acids and biogenic amines were quantified in positive mode. For the FIA‐MS/MS analysis, data were acquired using methanol as the running solvent, and acylcarnitines, lysophosphatidylcholines, phosphatidylcholines, and sphingomyelins were semi‐quantified. Evaluation of data quality was achieved by checking the accuracy and reproducibility of QC samples included in the p180 assay. Analysis was performed using MetaboAnalyst, where repeated *t*‐tests were used to compare normocapnic and hypercapnic treatments. Basal and LPS‐treated conditions were analyzed separately to avoid confounding effects introduced by LPS treatment. Metabolite data were normalized to the total metabolite sum prior to analysis. Multiple comparisons were controlled using a false discovery rate (FDR) correction, with an adjusted *p*‐value (FDR‐adjusted *p* < 0.05) considered statistically significant. Adjusted *p*‐values were calculated and used for subsequent statistical analyses.

LC–MS/MS summary data are included as Supplemental sheet 5 in Appendix S1.

### 2.13 Statistical Analysis

Unless otherwise stated, statistical analysis was performed using GraphPad Prism (Boston, MA, USA) (version8). ANOVA with appropriate recommended post hoc analysis or a two‐tailed *t*‐test was applied as indicated in the figure legends and with a level of significance set at *p* ≤ 0.05. Data are presented as mean ± standard deviation and shown in figures as histograms with error bars unless otherwise stated. A volcano plot represents data by displaying the −log_10_ adjusted *p*‐value on the y‐axis against the log_2_ fold change (log_2_FC) on the x‐axis. This allows simultaneous visualization of statistical significance and magnitude of change, enabling comparison between conditions such as 10% versus 5%. For all experiments, where indicated, independent experiments represent independent biological replicates (cell cultures independently passaged for a minimum of four passages) conducted on at least two separate days. LC–MS/MS data were analyzed using MetaboAnalyst, as described in the corresponding Methods section.

## 3 Results

### 3.1 Hypercapnia Disrupts ATP Content, NAD Species Abundance & Phospho‐ACC Expression

Our previous work indicated that THP‐1 cells exposed to hypercapnia demonstrate significant changes in genes associated with metabolism [12, 15]. However, how/if these changes in gene expression affect cellular metabolism and metabolites remains unclear. We found that hypercapnia significantly decreased total intracellular ATP levels following 24 h of exposure. Interestingly, while oligomycin—an ATP synthase inhibitor—reduced ATP levels relative to the vehicle control & significantly impaired cellular reductase activity (Figure S2E), hypercapnia induced a significant decrease in ATP, indicating a substantial disruption of cellular metabolic energetics (Figure 1A). Total NADH/NAD^+^ levels were also decreased in hypercapnia (Figure 1B). In addition, to account for the potential effects of hypercapnia on cell proliferation, viability or protein concentration and how these factors may influence total ATP and NADH/NAD^+^ abundance, we assessed these parameters following equal cell loading at time zero. After 24 h of hypercapnic exposure, cell proliferation, viability, and protein concentration were not affected in our monocytic model (Figure S2A–C).

**FIGURE 1:**
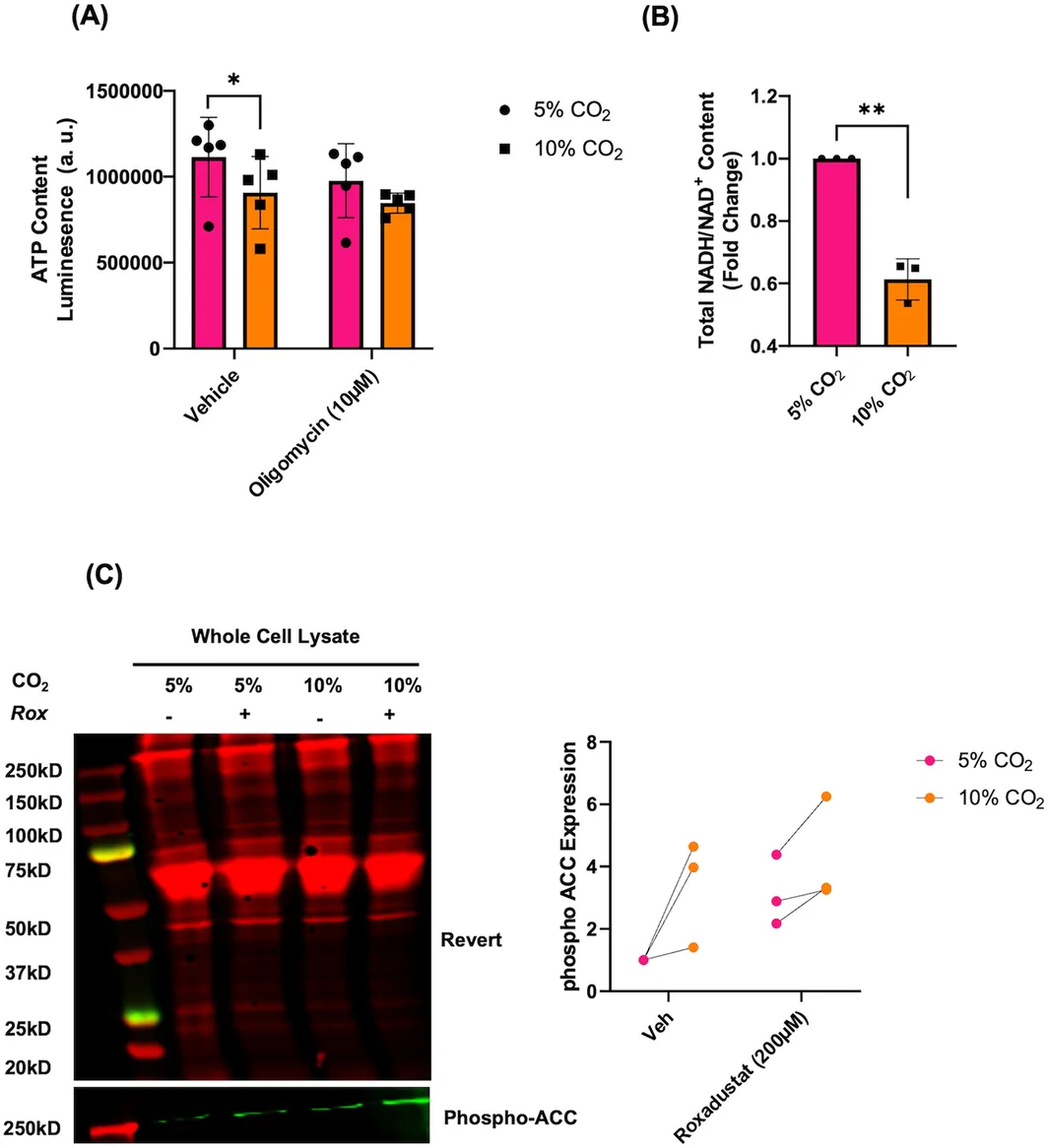
Hypercapnia disrupts phospho‐ACC expression, NAD species abundance, and ATP content. (A) ATP assay was performed on THP‐1 monocytes exposed to 5% or 10% CO_2_ ± oligomycin (10 μM) for 24 h. Absorbance was recorded using a CLARIOstar plate reader as per the methods. Data are representative of five independent experiments. Data shown are the mean values for each individual n‐number. (B) NADH/NAD^+^ assay was performed on THP‐1 monocytes exposed to 5% or 10% CO_2_ for 24 h. Absorbance was recorded using a CLARIOstar plate reader as per methods. Data are representative of three independent experiments. Data shown are the mean value for each individual n‐number. Statistical analysis was conducted using a paired *t*‐test (A) or repeated measures two‐way ANOVA, followed by Tukey's post hoc multiple comparisons test (B). Statistical significance is indicated as follows: *p < 0.05, **p < 0.01. (C) Western blot analysis of phospho‐ACC in THP‐1 whole‐cell lysates in the absence and presence of Roxadustat (200 μM, 20 h) during 24 h exposure to hypercapnia (*N* = 3). Lysates were probed using Revert total protein stain and imaged in the 700 nm channel. Blots were incubated with a primary phospho‐ACC antibody followed by a fluorescent secondary fluorophore‐conjugated antibody and imaged in the 800‐nm channel on an Li‐COR imaging system. Relative intensities were quantified using Empiria Studio 3.0 software. Data are representative of three independent experiments. ATP, adenosine triphosphate; NAD, nicotinamide adenine dinucleotide.

Given the established link between hypercapnia and lipid metabolism, specifically via the phospho‐ACC/CPT‐1α node [12, 14], we next examined whether 24‐h exposure to hypercapnia alters phospho‐ACC expression in THP‐1 monocytes. Hypercapnia enhanced the expression of phospho‐ACC (Ser79) (Figure 1C), demonstrating that the cells perceive hypercapnia as a robust metabolic stimulus. Elevated phospho‐ACC expression was observed regardless of the presence of Roxadustat. Roxadustat is a potent metabolic regulator via PHD inhibition and HIF‐1α stabilization (Figure S3), suggesting a robust and consistent hypercapnic effect on phospho‐ACC expression. Interestingly, in line with work previously published by Selfridge et al. [21], our findings demonstrate that hypercapnia reduces the expression of HIF‐1α in response to PHD inhibition (Figure S3). Taken together, these data demonstrate a clear hypercapnia‐dependent dysregulation of key bioenergetic components, which prompted us to further examine mitochondria in isolation under hypercapnic conditions.

### 3.2 Hypercapnia Disrupts Mitochondrial Function and Protein Content

Given the existing links between hypercapnic and mitochondrial adaptations, we next examined whether 24‐h hypercapnic exposure alters mitochondrial function in THP‐1 monocytes by assessing organelle characteristics, including structural membrane components, energetic status, and associated biochemical parameters. To assess characteristics of the mitochondrion, we measured mitochondrial membrane potential using BioTracker 405 (λEm 440 nm) and cardiolipin content using NAO (λEm 519 nm). FCCP is a mitochondrial uncoupling agent and is used here as a control to demonstrate the sensitivity of the BioTracker405 to changes in membrane potential (Figure 2A). While total BioTracker 405 fluorescence—indicative of overall mitochondrial membrane potential—was not significantly changed in hypercapnia (Figure 2A), NAO fluorescence was significantly decreased in hypercapnia (Figure 2B). Given that the accumulation of BioTracker 405 and other cationic‐based dyes are likely proportional to mitochondrial content, we attempted to control for this by calculating the BioTracker 405:NAO fluorescence ratio. This approach allows us to determine the CO_2_‐dependent effect on membrane potential relative to total mitochondrial content. Interestingly, the BioTracker 405:NAO fluorescence ratio was significantly increased, suggesting that hypercapnia increases mitochondrial membrane potential on a “per mitochondrion” basis when accounting for reduced cardiolipin levels (Figure 2C).

**FIGURE 2:**
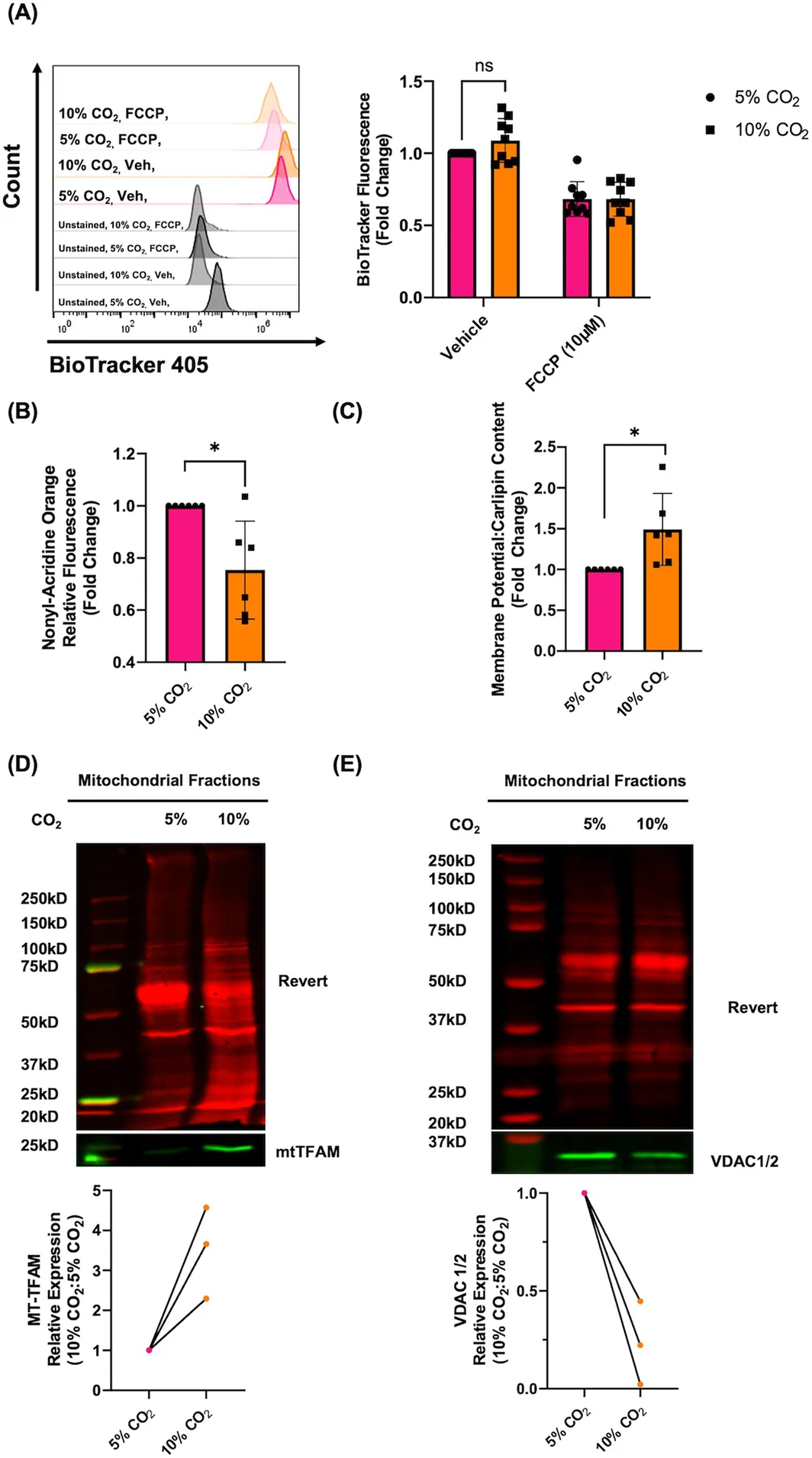
Hypercapnia disrupts mitochondrial function and protein content. (A) Representative flow cytometry histogram and corresponding quantification of THP‐1 monocytes stained with BioTracker405 ± 1 h of FCCP (10 μM) treatment following 24 h exposure to hypercapnia. (B) Flow cytometry analysis and corresponding quantifications were conducted to analyze cardiolipin content (Nonyl‐Acridine Orange, 520 ± 20 nm), in THP‐1 monocytes following 24 h exposure to hypercapnia. (C) Normalization and quantification of mitochondrial membrane potential (BitoTracker405, 440 ± 20 nm) and cardiolipin content (Nonyl‐Acridine Orange, 520 ± 20 nm) in THP‐1 monocytes following 24 h exposure to hypercapnia. Flow cytometry was conducted using a Beckman Coulter CytoFlex LX. Data represent six independent experiments (*N* = 6) and are presented as geometric mean ± standard deviation unless otherwise stated. Statistical analysis was conducted using repeated measures two‐way ANOVA, followed by Tukey's post hoc multiple comparisons test (A) or a paired *t*‐test (B, C). Statistical significance is indicated as follows: *p < 0.05. Western blot analysis of (D) TFAM and (E) VDAC expression in THP‐1 monocytes following 24 h exposure to hypercapnia (*N* = 3). Western blot analysis of lysates were probed using Revert total protein stain and imaged in the 700 nm channel. Blots were incubated with a TFAM or VDAC1/2 primary antibody followed by a fluorescent secondary fluorophore‐conjugated antibody and imaged in the 800‐nm channel on a Li‐COR imaging system. Relative intensities were quantified using Empiria Studio 3.0 Software.

Additionally, we assessed TFAM, a key regulator of mitochondrial transcription, and VDAC, a principal mitochondrial metabolite transporter. Western blot analysis of mitochondrially enriched fractions revealed that hypercapnia significantly increased mitochondrial‐associated TFAM while decreasing mitochondrial‐associated VDAC expression (Figure 2D,E), indicating that elevated CO_2_ alters mitochondrial–DNA transcription factors and mitochondrial transporter expression.

### 3.3 LC–MS/MS Analysis Revealed Hypercapnic‐Induced Changes in Lipid Abundance

We next employed liquid chromatography–mass spectrometry (LC–MS/MS) to specifically investigate the metabolic consequences of 24‐h hypercapnic exposure on THP‐1 monocytes with or without 22 h LPS stimulation using a targeted metabolomic analysis (Figure 3A,B, Figures S4 and S5). LPS treatment was included solely as a robust metabolic perturbation control to identify the most pronounced metabolic alterations associated with hypercapnia. Metabolite identification and downstream analyses were specifically directed toward distinguishing hypercapnia‐dependent effects from those driven by LPS treatment. Additional heat map analyses of LPS‐dependent metabolic alterations are presented in Figures [Link], [Link], [Link]. Following *t*‐test analysis of both basal and LPS‐stimulated conditions, among the commonly (i.e., significant in both basal & LPS) detected lipid species, only two (C4 and LysoPCaC18:0) were consistently decreased under both conditions. In contrast, a total of 32 lipid species were significantly increased under hypercapnia in both states, indicating that elevated CO_2_ exposure drives consistent and pronounced shifts in lipid metabolism in monocytes (Figure 3C–F, Figures S9 and S10). Aligning with our findings of hypercapnia and phospho‐ACC expression, we observe that hypercapnia induces a complex alteration of lipid abundance, affecting both short‐ and long‐chain carnitines, phosphatidylcholines, and sphingomyelins, which are predominantly increased. Specifically, we identify an increase in a broad lipid signature comprising multiple phosphatidylcholines (both diacyl PC aa and ether‐linked PC ae species spanning C30–C42), selected lysophosphatidylcholines (LysoPC a C16:1, C28:0, C28:1), sphingomyelins (SM C16:1, C18:1, (OH) C22:2), and acylcarnitines (C2, C5, C5‐OH (C3‐DC‐M), C14). Collectively, these changes indicate a coordinated increase in lipid species in monocytes under hypercapnic conditions.

**FIGURE 3:**
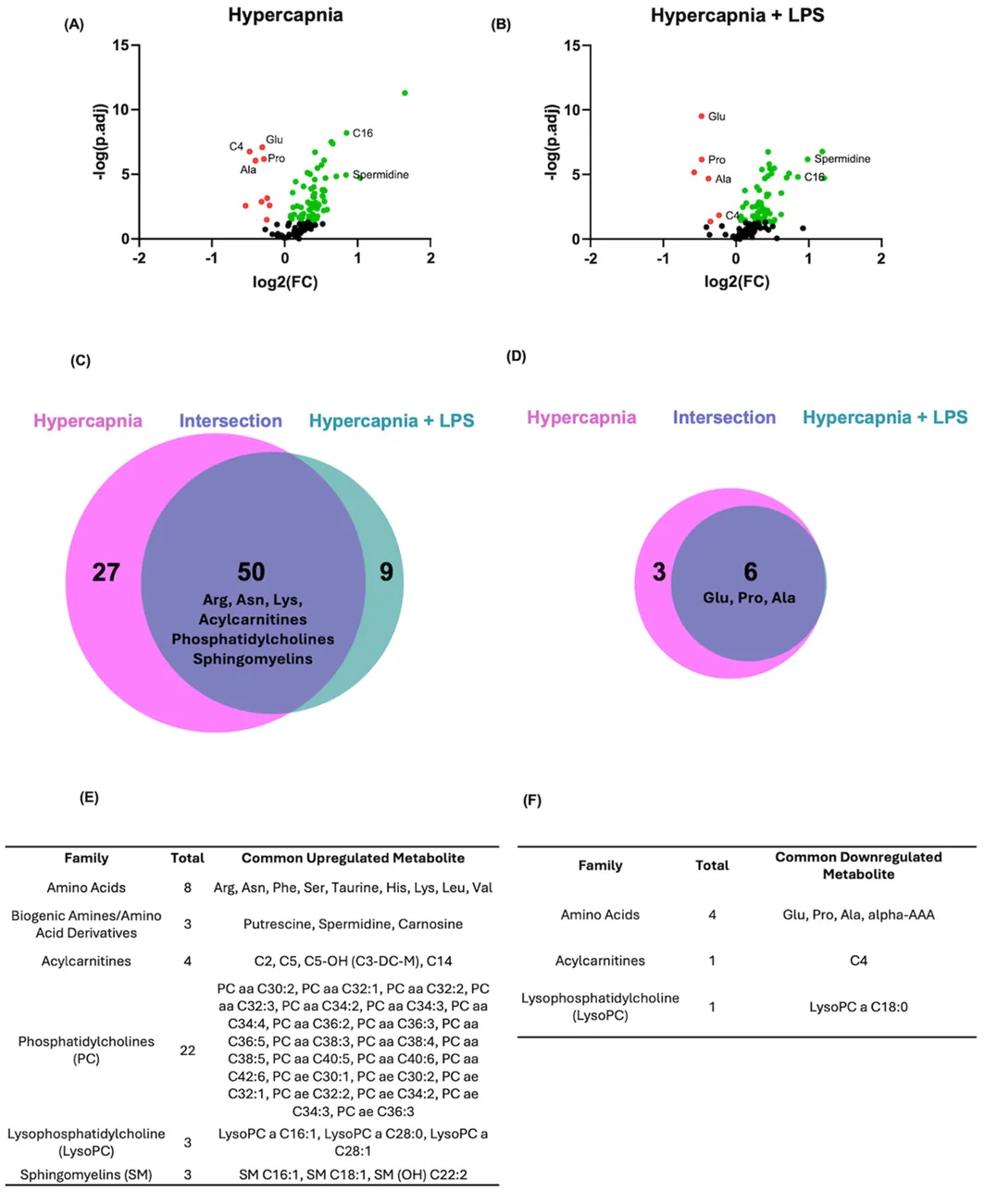
LC–MS/MS metabolomic analysis reveals that hypercapnia significantly alters amino acid and lipid species abundance in THP‐1 monocytes. Volcano plot analysis of metabolites in the (A) absence and (B) presence of LPS (2.5 μg mL^−1^ for 22 h) during 24 h exposure to hypercapnia. The cutoff for significance is *p*‐adj < 0.05. Significantly increased metabolites are shown in green, and decreased metabolites are shown in red. Venn diagram representing the number of metabolites that are commonly (C) increased and (D) decreased metabolites in the absence (pink) and presence (teal) of LPS (2.5 μg mL^−1^ for 22 h) following 24 h exposure to hypercapnia. Tabled representation of (E) increased and (F) decreased amino acids and lipid species derived from the intersection of Venn diagrams 4C and 4D. Data represent 8–10 independent experiments (*N* = 8–10).

### 3.4 LC–MS/MS Profiling Identifies Hypercapnia‐Driven Remodeling of Amino Acid Metabolism

We next focused our attention on the more novel and relatively unexplored effects of CO_2_ on amino acid metabolism. LC–MS/MS analysis of THP‐1 cells exposed to hypercapnia demonstrated two patterns of altered amino acids (Figure 4A). The decreased pattern consisted primarily of nonessential amino acids linked to glutaminolysis, transamination, and mitochondrial carbon metabolism (glutamate, proline, alanine, as well as aspartate (under basal conditions only)). This pattern may suggest dysregulation of the early reactions in the glutaminolysis pathway. Glutamine serves as the key amino and carbon donor preceding glutamate synthesis (Figure 4B), functioning as a primary substrate that fuels mitochondrial biosynthetic processes. While intracellular glutamine levels remained unchanged under hypercapnic conditions, LC–MS/MS analysis notably revealed significant reductions in downstream amino acids—including glutamate, aspartate, proline, and alanine (Figure 4C,E). Importantly, the levels of most of these amino acids (except aspartate) were reduced under hypercapnia in both basal‐ and LPS‐stimulated states, indicating a robust response to CO_2_. Aspartate and alanine are direct products of transaminase reactions in which glutamate serves as the main amino donor, whereas proline synthesis is initiated from glutamate as its primary precursor (not via transamination) (Figure 4B,D). Lastly, we interestingly observed that analysis of the effects of LPS alone on metabolite abundance identified 24 independently regulated metabolites whose abundance increased following LPS exposure. These 24 metabolites accounted for 77% of the total significantly upregulated metabolites. Notably, none of these LPS‐induced changes were significant under hypercapnic conditions, suggesting that elevated CO_2_ elicits an alternative metabolic response (Figure S8). Collectively, these observations strongly suggest for the first time that hypercapnia affects mitochondrial‐dependent processes that rely on glutamine and glutamate as central carbon and nitrogen substrates.

**FIGURE 4:**
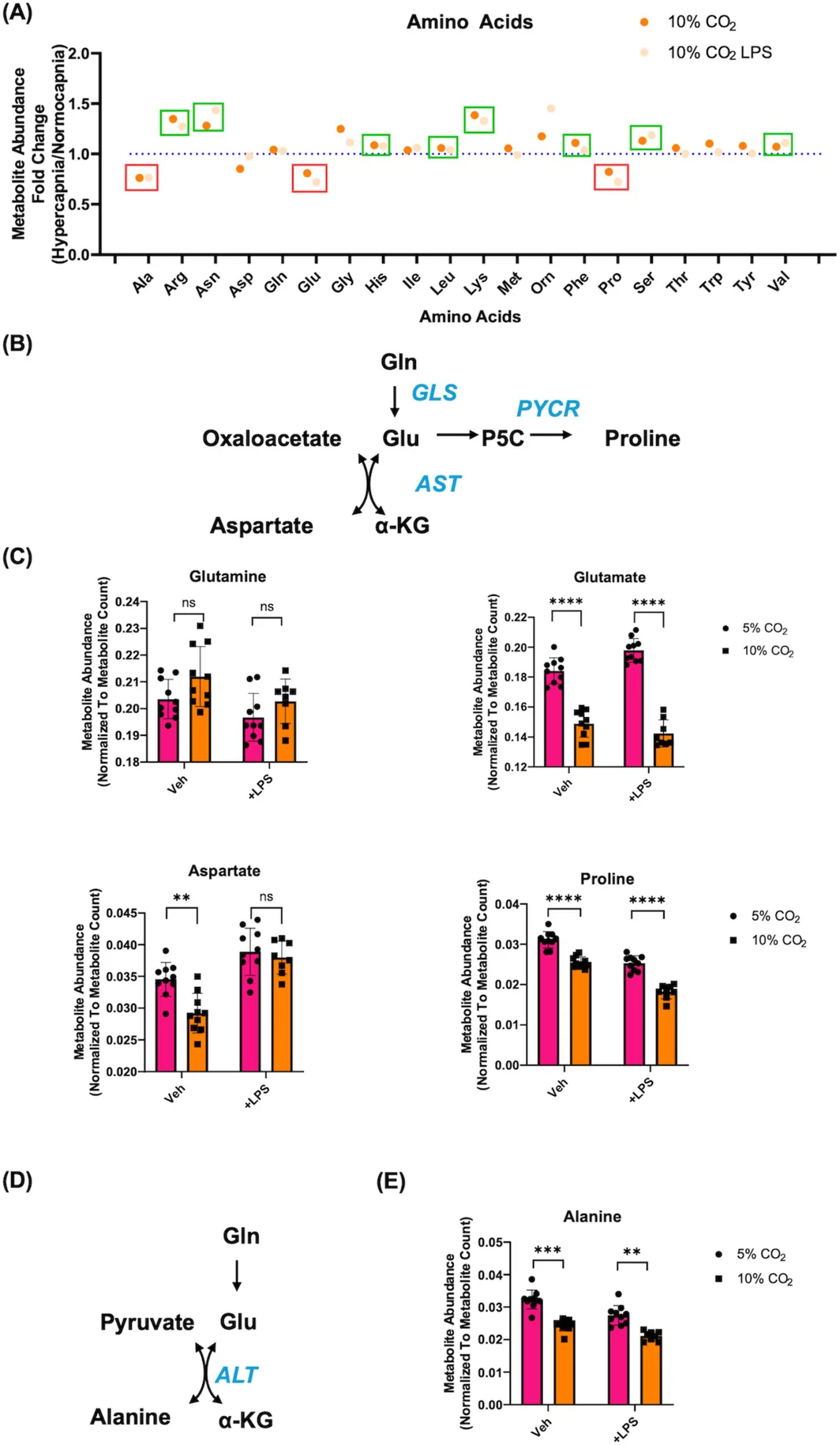
LC–MS/MS analysis reveals that hypercapnia significantly alters the glutaminolysis pathway in the presence and absence of LPS. (A) Visual summary of data derived from Figure 3 focusing on amino acid abundance in THP‐1 cells exposed to 5% or 10% CO_2_ for 24 h. Samples exposed to 10% CO_2_ are colored orange, and 10% CO_2_ + LPS are colored light orange. Data are presented as fold change relative to the corresponding 5% CO_2_ control and compiled from 10 independent experiments. Statistically significant changes observed in both basal and LPS‐stimulated states are highlighted using grouped boxes: Green indicates increase abundance, whereas red indicates decreased abundance. No change (fold change = 1) is represented by a blue dashed line, corresponding to the 5% CO_2_ abundance baseline. (B) Schematic diagram depicting the canonical pathway of glutamine entry to the TCA cycle. (C) Quantification of metabolite abundance (normalized to metabolite count) for glutamine, glutamate, aspartate, and proline in the absence and presence of LPS (2.5 μg mL^−1^ for 22 h) during 24 h exposure to hypercapnia. (D) Schematic diagram depicting the canonical pathway of glutamine‐dependent synthesis of alanine and alpha‐ketoglutarate. (E) Quantification of metabolite abundance (normalized to metabolite count) of alanine in the absence and presence of LPS (2.5 μg mL^−1^ for 22 h) during 24 h exposure to hypercapnia. Data represent 8–10 independent experiments (*N* = 8–10) and are presented as mean ± standard deviation. Statistical analysis was conducted using repeated measures two‐way ANOVA, followed by Tukey's post hoc multiple comparisons test. Statistical significance is indicated as follows: **p < 0.01, ***p < 0.001, ****p < 0.0001. AST, aspartate transaminase; GLS1, glutaminase 1; PYCR1, pyrroline‐5‐carboxylate reductase 1.

### 3.5 GC–MS Analysis Reveals Hypercapnia Altered Amino Acid and TCA Cycle Metabolite Abundance

Based on the findings above, we next investigated glutamate utilization using culture medium supplemented with uniformly labeled U‐[^13^C]glutamine. Gas chromatography–mass spectrometry (GC–MS) then determined the effect of hypercapnia on glutamine utilization in THP‐1 monocytes. In addition to the findings reported from our LC–MS/MS analysis, the GC–MS assay platform captured metabolites associated with the glycolytic pathway, the TCA cycle, and other mitochondrial intermediates, some of which overlap and some of which are unique relative to the metabolites shown in Figures 3 and 4. Initial analysis of total intracellular metabolites revealed that hypercapnia significantly altered the concentrations of amino acids (aspartate, glutamate, proline) as well as TCA cycle intermediates (succinate, malate) (Figure 5A,B). The most pronounced hypercapnia‐dependent effects were observed in the abundances of aspartate, succinate, proline, and glutamate. This approach, in distinct biological experiments, confirms and validates our conclusions from the LC/MS/MS experiments—that hypercapnia alters the levels of key amino acids. Furthermore, the GC–MS approach reveals additional hypercapnia‐sensitive metabolites that which were not part of the LC–MS/MS screen, for example—succinate, malate, and glycerol‐3‐phosphate (Glyc3P). Collectively, these results reinforce the hypothesis that elevated CO_2_ disrupts glutamine metabolism and associated mitochondrial‐dependent metabolic pathways including the TCA cycle.

**FIGURE 5:**
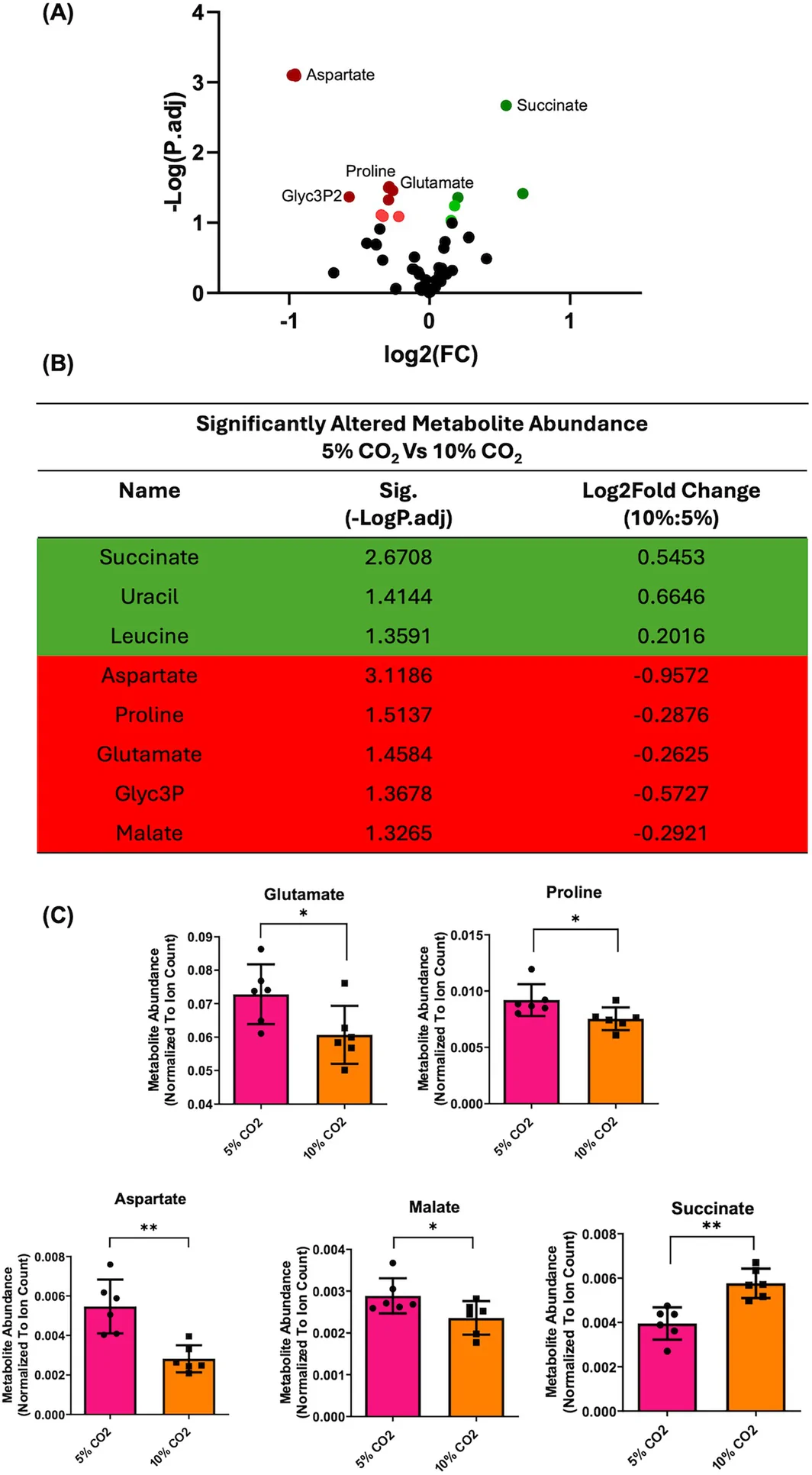
GC–MS analysis reveals that hypercapnia significantly alters the amino acid and TCA cycle metabolites abundance. (A) Volcano plot analysis of significantly upregulated and downregulated metabolites following 24 h exposure to hypercapnia. The cutoff for significance is *p*‐adj < 0.05. Significantly (*p*‐adj < 0.05) increased metabolites are shown in green, and decreased metabolites are shown in red, whereas modestly (0.05 < *p*‐adj < 0.1) increased metabolites are shown in light green and modestly decreased metabolites are shown in light red. (B) Tabled *p*‐adj values of amino acids and TCA cycle metabolites altered following 24 h exposure to hypercapnia. Increased metabolites are shown in green, and decreased metabolites are shown in red. (C) Quantification of amino acid and TCA cycle metabolite abundances (normalized to ion count) following 24 h exposure to hypercapnia. Data represent six independent experiments (*N* = 6) and are presented as mean ± standard deviation. Statistical analysis was conducted using a paired t‐test. Statistical significance is indicated as follows: *p < 0.05, **p < 0.01.

### 3.6 GC–MS Analysis Reveals Hypercapnia Alters U‐[ ^13^C]Glutamine Utilization in THP‐1 Monocytes

Using GC–MS, we next traced U‐[^13^C]glutamine utilization through key metabolic pathways (Figure 6). Interestingly, although total cellular uptake of U‐[^13^C]glutamine was unaffected, we observed a significant reduction in ^13^C enrichment of intracellular glutamate (M + 5 isotopologue) under hypercapnic conditions (Figure 6B,C, Figure S11). This suggests that glutamine uptake by the cells was unchanged by hypercapnia but that there is significantly altered conversion of glutamine to glutamate—the first enzyme reaction of the glutaminolysis pathway as evidenced by the reduction in the M + 5 isotopologue. Correspondingly, a marked reduction in total ^13^C labeling of downstream metabolites was observed, suggesting a diminished incorporation of glutamine‐derived carbon into mitochondrial biosynthetic pathways (Figure 7B). Hypercapnia exerted the most pronounced effect on U‐[^13^C]glutamine incorporation into aspartate (M + 4 isotopologue) (Figure 7B).

**FIGURE 6:**
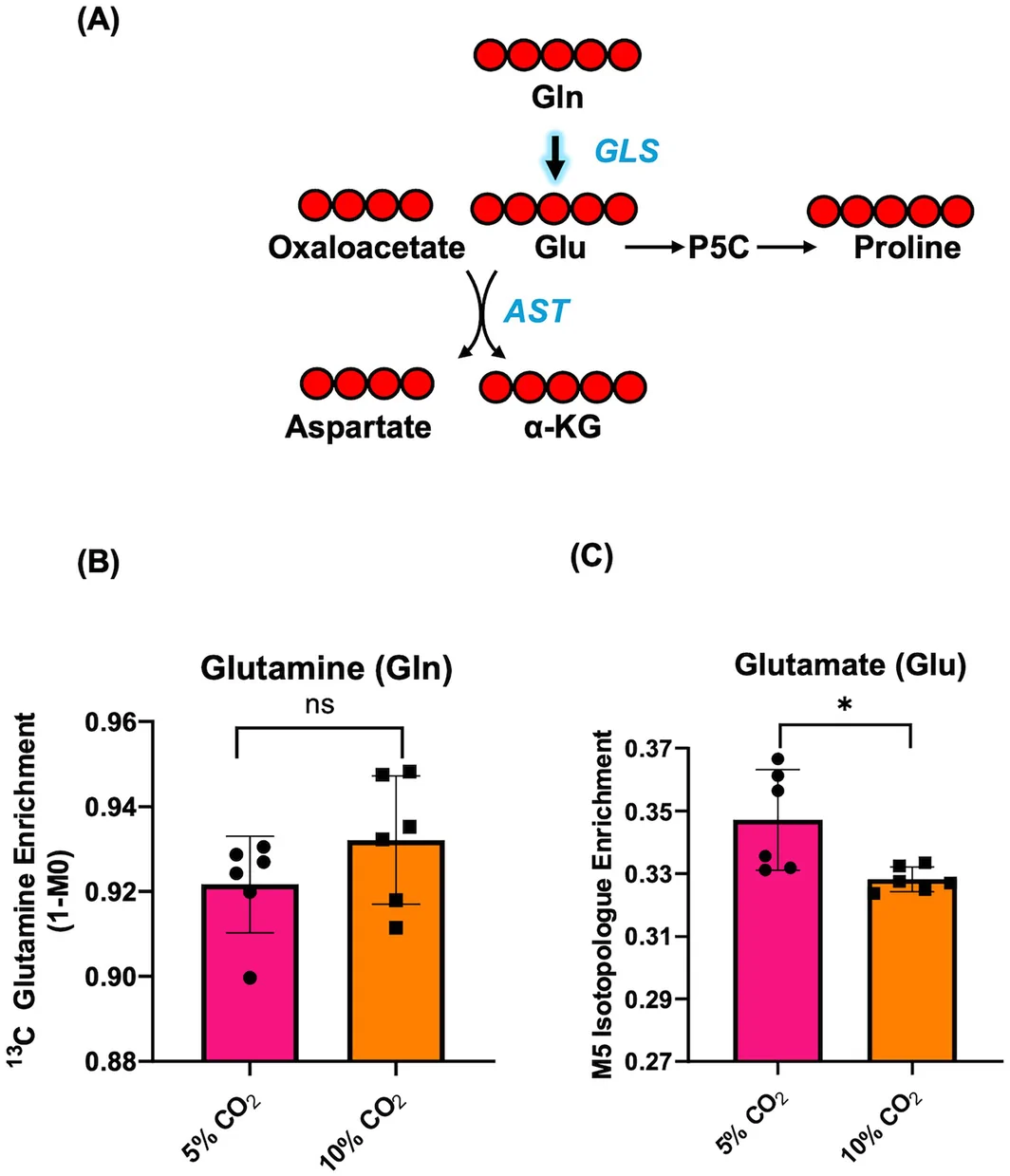
GC–MS analysis reveals that hypercapnia significantly alters U‐[^13^C]glutamine enrichment of amino acids. (A) Schematic diagram depicting the canonical pathway and carbon distribution of U‐[^13^C]glutamine enrichment of amino acids. Specific nodes of focus are indicated by blue glowing arrows. Quantification of U‐[^13^C]glutamine enrichment of (B) glutamine (Gln) & (C) glutamate (Glu) following 24 h exposure to hypercapnia. Data analyzed represent either the total U‐[^13^C] glutamine enrichment quantification or analysis of individual isotopologues (Y‐axis). Data represent six independent experiments (*N* = 6) and are presented as mean ± standard deviation. Statistical analysis was conducted using a paired *t*‐test. Statistical significance is indicated as follows: *p < 0.05. AST, aspartate transaminase; GLS1, glutaminase 1.

**FIGURE 7:**
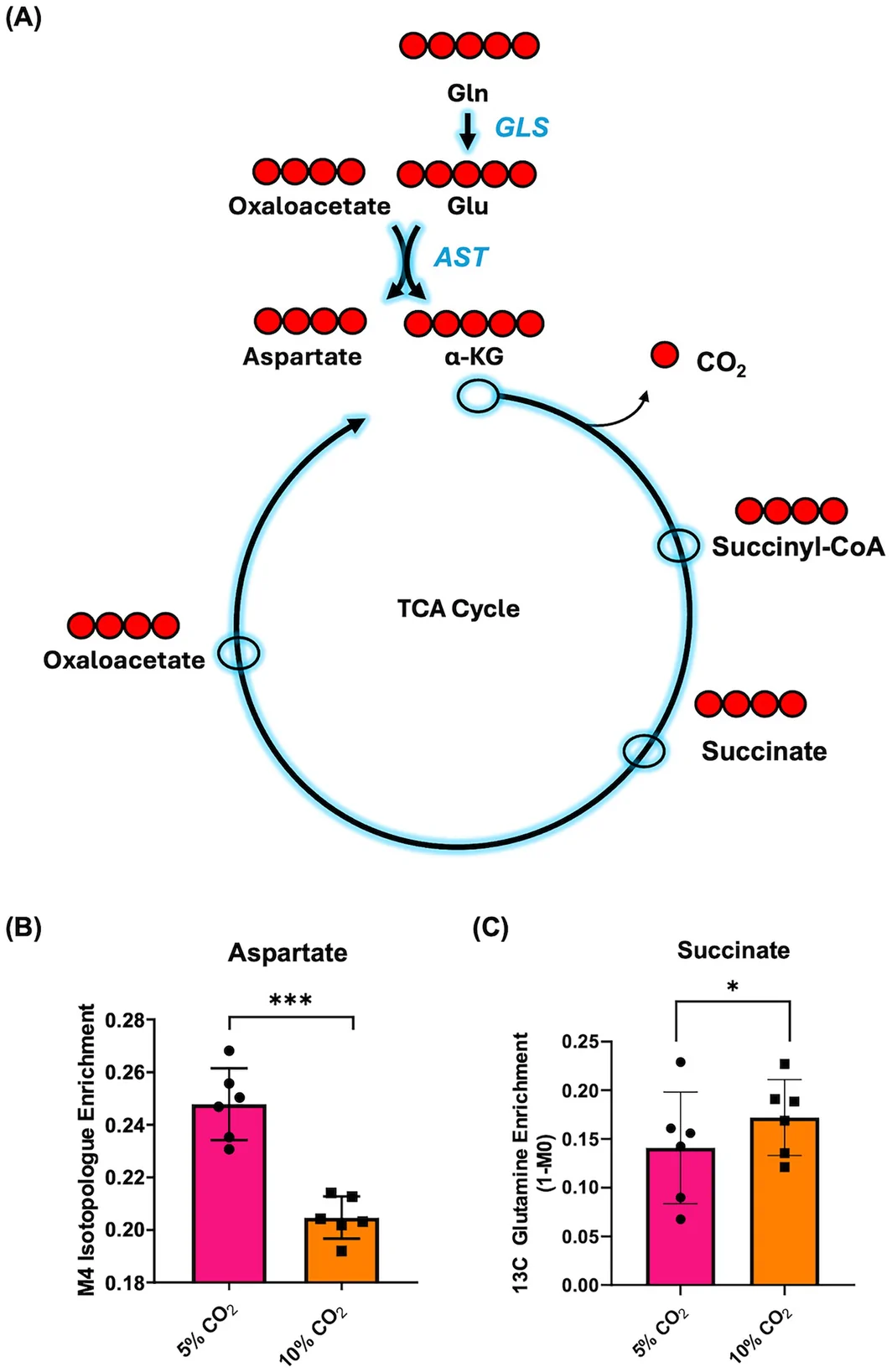
GC–MS analysis reveals that hypercapnia significantly alters U‐[^13^C]glutamine enrichment of amino acids and TCA cycle metabolites. (A) Schematic diagram depicting the canonical pathway and carbon distribution derived from U‐[^13^C]glutamine enrichment of TCA cycle metabolites. Specific nodes of focus are indicated by blue glowing arrows. (B) Quantification of U‐[^13^C]glutamine enrichment of (B) aspartate and (C) succinate following 24 h exposure to hypercapnia. Data analyzed represent the total U‐[^13^C]glutamine enrichment or analysis of individual isotopologues (Y‐axis). Data represent six independent experiments (*N* = 6) and are presented as mean ± standard deviation. Statistical analysis was conducted using a paired *t*‐test. Statistical significance is indicated as follows: *p < 0.05, ***p < 0.001. AST, aspartate transaminase; GLS1, glutaminase 1; PYCR1, pyrroline‐5‐carboxylate reductase 1; TCA cycle, tricarboxylic acid cycle.

Given the observed alterations in U‐[^13^C]glutamine enrichment of glutamate and aspartate, these data suggest a hypercapnic‐dependent adaptation alters U‐[^13^C]glutamine flux into the mitochondria—that is, M + 4 aspartate is derived from M + 4 oxaloacetate during transamination. This would indicate that full TCA cycle utilization of U‐[^13^C]glutamine is decreased. This could imply that hypercapnia may affect the conversion of glutamine to glutamate and/or induce more widespread adaptations in glutamine metabolism and mitochondrial processing. Collectively, these findings indicate that hypercapnia alters glutamine utilization, thereby limiting its contribution to anabolic pathways.

### 3.7 GC–MS Analysis Reveals Hypercapnia Alters U‐[ ^13^C]Glucose Enrichment of Glycolysis and TCA Cycle in THP‐1 Monocytes

Given that U‐[^13^C]glutamine tracing revealed glutamine incorporation accounted for less than half of the carbon contribution to key metabolic intermediates, it is likely that other carbon‐rich nutrients contributed to the synthesis of the metabolites detected in our screen. As glucose represents a primary cellular carbon source, metabolized through glycolysis to generate pyruvate for TCA cycle entry, we next sought to determine whether hypercapnia could also alter glucose metabolism using U‐[^13^C]glucose incorporation.

GC–MS analysis revealed that hypercapnia significantly decreased the U‐[^13^C]glucose enrichment of M + 3 Glycerol‐3‐phosphate (Glyc3P), a key intermediate in the triacylglycerol synthesis pathway, which branches directly from the glycolytic pathway (Figure S12B). Moreover, hypercapnia elicited mild reductions in total U‐[^13^C]glucose enrichment of lactate (Figure S12C). Interestingly, U‐[^13^C]glucose enrichment of the pyruvate M + 3 isotopologue remained unchanged (Figure S12D), however, hypercapnia significantly reduced U‐[^13^C]glucose incorporation into M + 3 alanine—a gluconeogenic amino acid generated via alanine transaminase, which uses glutamate and pyruvate as precursor substrates (Figure S12E). In relation to TCA cycle intermediates, we observed mean increases in isotopologues of α‐KG, succinate, and malate; however, they fell just short of statistical significance (Figure S13B–D). Thus, there is some evidence to suggest that hypercapnia enhances the flow of glucose‐derived carbons into the TCA cycle while concurrently suppressing amino acid biosynthesis. However, our data indicate a more robust and prominent role for altered glutamine metabolism compared to glucose metabolism under these particular experimental conditions.

### 3.8 Hypercapnia Dysregulates the Glutamate and Proline Synthesis Pathway

To further elucidate the mechanistic and functional consequences of altered glutamine metabolism under hypercapnia, we next focused on the glutamine‐proline biosynthesis axis (Figure 8A). Glutamine serves as a key amino and carbon donor for proline synthesis, primarily through its conversion to glutamate and subsequent reduction to pyrroline‐5‐carboxylate (P5C), which is then converted to proline by the mitochondrial enzyme pyrroline‐5‐carboxylate reductase 1 (PYCR1) (Figure 8A). PYCR1 plays a vital role in maintaining redox homeostasis and supporting anabolic metabolism. The deployment of LC–MS/MS, GC–MS, and U‐[^13^C]glutamine applications identified that hypercapnia significantly reduces both glutamate and proline abundance (Figures 4 and 5) alongside a significant reduction of U‐[^13^C]glutamine incorporation into glutamate and proline, respectively (Figures 6C and 8E). To reiterate, we did not observe any alterations in intracellular glutamine abundance, indicating that basal glutamine availability is unlikely to be a driving factor in this process. To gain mechanistic insights into the CO_2_‐dependent adaptation of this pathway, we next examined whether 24‐h exposure to hypercapnia similarly affects mitochondrial GLS‐1 and PYCR1 protein expression using mitochondrial‐enriched fractions. Our findings confirm a hypercapnia‐dependent modulation of this biosynthetic axis, with mitochondrial expression of GLS1 upregulated and PYCR1 downregulated (Figure 8C,F). This pattern highlights a significant adaptation at key metabolic checkpoints within glutaminolysis and other biosynthetic pathways that give mechanistic insight into the observed effects of hypercapnia on amino acid metabolism.

**FIGURE 8:**
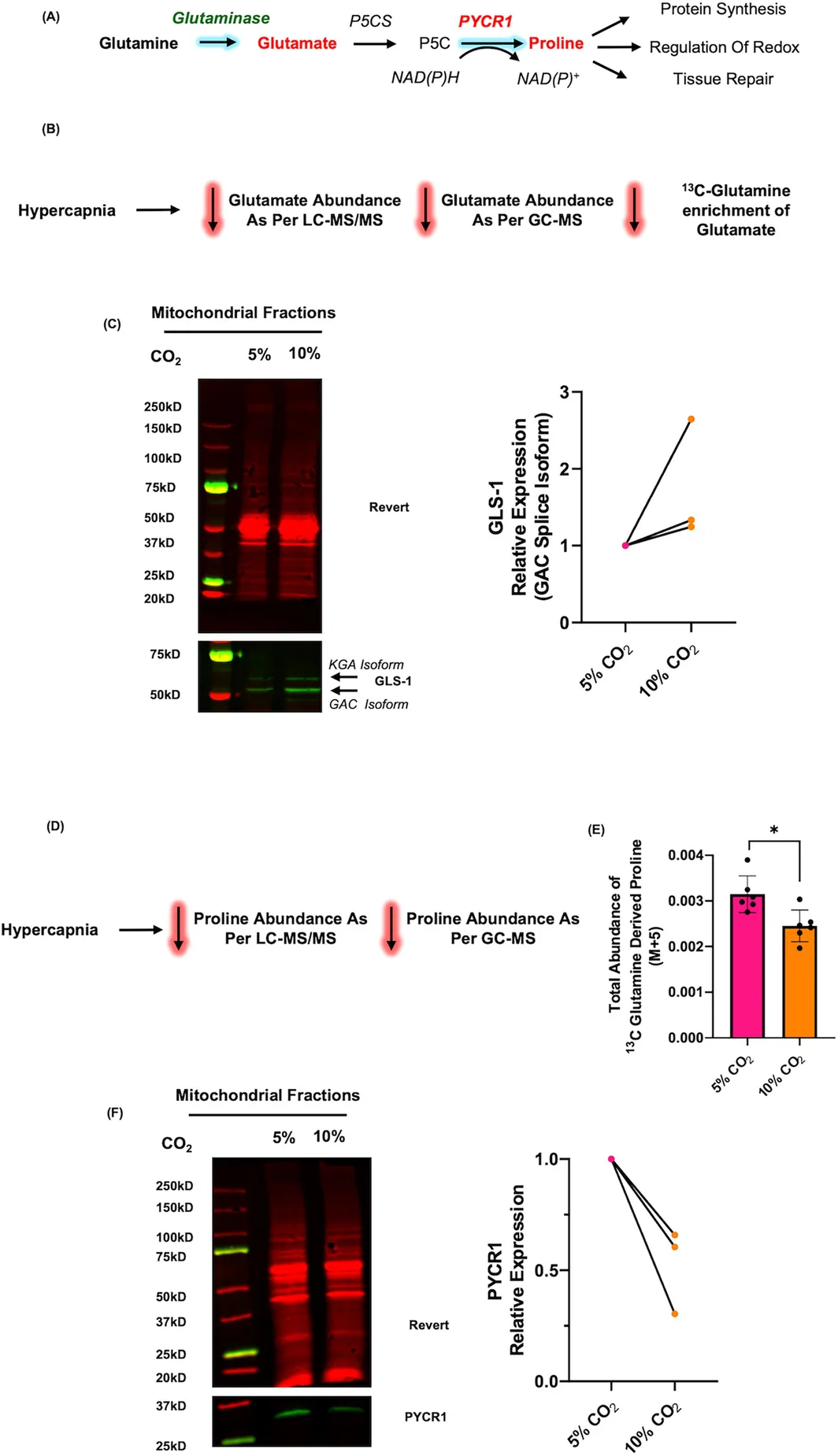
Hypercapnia dysregulates proline synthesis in THP‐1 monocytes. (A) Schematic diagram depicting the canonical pathway, whereby glutamine is the primary amino and carbon source for proline synthesis. Specific nodes of focus are indicated by blue glowing arrows. Proteins and metabolites that are increased colored green, those that are decreased are colored red. (B) Schematic depicting our findings on how hypercapnia affects glutamate abundance, measured by LC–MS/MS and GC–MS, as well as ^13^C‐glutamine enrichment into glutamate. (C) Western blot analysis of mitochondrial GLS‐1 expression in THP‐1 monocytes following 24 h exposure to hypercapnia (*N* = 3). Lysates were probed using Revert total protein stain and imaged in the 700 nm channel. Blots were incubated with a GLS‐1 primary antibody followed by a fluorescent secondary fluorophore‐conjugated antibody and imaged in the 800‐nm channel on an Li‐COR imaging system. Relative intensities were quantified using Empiria Studio 3.0 Software. (D) Schematic depicting our findings on how hypercapnia affects proline abundance, measured by LC–MS/MS and GC–MS. (E) Quantification of U‐[^13^C]glutamine enrichment of the M + 5 isotopologue of proline following 24 h exposure to hypercapnia. Data were generated by multiplying isotopologue labeling by metabolite abundance to represent the total abundance of ^13^C‐glutamine‐derived amino acids. This approach accounts for differences in overall amino acid levels between conditions when assessing label incorporation. (F) Western blot analysis of mitochondrial PYCR1 expression in THP‐1 monocytes following 24 h exposure to hypercapnia (*N* = 3). Lysates were probed using Revert total protein stain and imaged in the 700 nm channel. Blots were incubated with a PYCR1 primary antibody followed by a fluorescent secondary fluorophore‐conjugated antibody and imaged in the 800‐nm channel on an Li‐COR imaging system. Relative intensities were quantified using Empiria Studio 3.0 Software. Statistical analysis was conducted using repeated measures two‐way ANOVA, followed by Tukey's post hoc multiple comparisons test (B, E) or a pair *t*‐test (C, D). Statistical significance is indicated as follows: *p < 0.05. GLS1, glutaminase 1; P5CS, Δ^1^‐pyrroline‐5‐carboxylate synthetase; PYCR1, pyrroline‐5‐carboxylate reductase 1.

## 4 Discussion

Here, we demonstrate how hypercapnia alters the metabolic preferences of human THP‐1 monocytes. In a mammalian physiological and pathophysiological context, these findings may offer insight into the downstream consequences in patients suffering from systemic hypercapnia. By tracking metabolic and bioenergetic profiles, we observe a hypercapnia‐induced mitochondrial adaptation that leads to alterations in baseline protein expression and metabolite abundance. Unlike the well‐established hypoxia field, where the consequences of microenvironmental changes in oxygen have been carefully linked to altered metabolism, cellular phenotype, and physiological function, the effect of CO_2_ on metabolism is much less well understood. This study directly addresses this research gap by providing robust supportive data in support of a hypercapnia‐dependent metabolic phenotype using a range of targeted biochemical and unbiased metabolomic approaches. Taken together, these data provide evidence of specific changes in lipid and amino acid metabolism that are distinct from metabolic responses to hypoxia and clearly point to altered glutamine processing in hypercapnia.

Over the past two decades, increasing emphasis on mitochondrial function has underscored the essential role of this organelle in regulating central aspects of cellular activity, including immune cell activation, phenotypic differentiation, tissue remodeling, and effector responses [22]. With that said, a body of work positions hypercapnia as a potent stimulus that alters mitochondrial function [11, 12]. We first observed that the content of intracellular ATP and total NADH/NAD^+^ species was significantly reduced under hypercapnic conditions (Figure 1A,B), suggesting that elevated CO_2_ has a pronounced effect on cellular energetics. This observation is further supported by an increase in phospho‐ACC in hypercapnia in cells with and without Roxadustat treatment (a positive control for catabolic signaling) (Figure 1C). Interestingly, in line with previous work [21], we further observed a hypercapnia‐dependent reduction of HIF‐1α expression in response to pharmacological PHD inhibition (Figure S3). Moreover, the reduction in ATP is consistent with the findings of Vohwinkel et al. in N12 lung fibroblasts and A549 lung epithelial cells exposed to hypercapnia for 1 and 3 days [11]. Interestingly, our observed changes in phospho‐ACC (and lipid metabolism) in THP‐1 monocytes are evident at 24 h and 10% CO_2_, which is earlier and at a more modest degree of hypercapnia compared to the observations of Ceco et al. (exposing myoblasts for 3 days to 15% CO_2_) [14]. Taken together, our data in THP‐1 monocytes are congruent with findings in a range of other cells which is suggestive of a general hypercapnia‐dependent adaptation of mitochondrial metabolism.

Employing a flow cytometry approach, we observed that hypercapnia induces a marked reduction in cardiolipin content [18] but an increase in ΔΨm on a “per mitochondrion” basis (Figure 2C). While cardiolipin is required for optimal activity of inner mitochondrial membrane proteins, particularly those of the electron transport chain via complex formation [23], it may be evident that cardiolipin loss (indicating reductions in mitochondrial mass or number) may disrupt mitochondrial integrity and impair electron transport chain efficiency. The observed elevations of ΔΨm may serve as a compensatory mechanism to counteract declining mitochondrial output (or lack of regulatory control). It remains unclear whether the observed changes in cardiolipin content and ΔΨm contribute directly to the hypercapnia‐induced reduction in ATP synthesis. While we speculate that these alterations may represent a compensatory mitochondrial response, this adaptation may be insufficient to maintain basal ATP production. Studying mitochondrial‐enriched cellular fractions, we revealed that hypercapnia led to increased expression of mt‐TFAM, which is essential for mitochondrial DNA transcription and biogenesis [24]. Hypercapnia, however, reduces the expression of mt‐VDAC, a key mitochondrial membrane transporter [24, 25], further supporting the hypothesis that hypercapnia alters basal mitochondrial homeostasis (Figure 2D,E). Both our data and those of Phelan et al. [12] may suggest that the transcriptional upregulation of mitochondrial subunits represents an adaptive response to hypercapnic stress (via TFAM expression and mt‐*mRNA* abundance), occurring as early as 4 h and persisting up to 24 h. Despite these adaptive responses, it is important to reiterate that we observed a significant reduction in overall mitochondrial mass. These data suggest that there is significant remodeling of mitochondrial function in response to hypercapnia.

To capture the metabolic landscape of hypercapnic immune cells, we conducted an LC–MS/MS‐targeted screen to assess changes in metabolite levels under elevated CO_2_ conditions. Twenty‐four‐hour exposure to hypercapnia significantly increased levels of several acylcarnitine species compared to normocapnia—aligning with previous studies conducted in THP‐1 monocyte models as well as skeletal muscle models [12, 14] suggesting that the observed changes may be a general response to hypercapnia and not restricted to THP‐1 or indeed immune cells. Our LC–MS/MS analysis revealed a hypercapnia‐dependent increase in several lipid species (Figure 3), notably acylcarnitine abundance, which bypasses the malonyl‐CoA:CPT‐1α regulatory checkpoint to facilitate fatty acid transport into the mitochondrial matrix. As mentioned above, we identified that hypercapnia induces the upregulation of phospho‐ACC (Figure 1C), which inhibits the conversion of acetyl‐CoA to malonyl‐CoA. Malonyl‐CoA is a key allosteric inhibitor of mitochondrial fatty acid transport via CPT‐1α [26] and participates in lipogenesis. This phospho‐ACC activation is indicative of a metabolic phenotype preferring catabolic processes promoting mitochondrial β‐oxidation. These findings are also in line with a more brief (4‐h) hypercapnic exposure, which significantly altered the abundance of several mitochondrial acylcarnitine species and genes associated with β‐oxidation [12]. In addition, while hypercapnia is generally considered immunosuppressive, our findings suggest that hypercapnia may, at least in part, perturb the metabolic response to LPS stimulation. Analysis of LPS‐induced changes in metabolite abundance identified 24 independently regulated metabolites that were significantly increased following LPS exposure, accounting for 77% of the total upregulated metabolites observed. Strikingly, none of these metabolite changes were detected under hypercapnic conditions, suggesting that elevated CO_2_ may fundamentally alter the metabolic response following inflammatory stimulation (Figure S8). Collectively, by reshaping key metabolic pathways, elevated CO_2_ drives a unique metabolic adaptation which may set the stage for broader effector function consequences.

While the effects of hypercapnia on lipid metabolism have been partially characterized previously, there are very few studies, to our knowledge, that have focused on alterations in amino acid metabolism. One noteworthy study reported that hypercapnia significantly dysregulated metabolites involved in l‐glutamate pathways and transport, as well as amino acid metabolic pathways associated with alanine, glycine, and cysteine. Amino acid metabolism, however, was not the focus of that study [14]. In our models, hypercapnia specifically decreased glutamate, proline, alanine, and aspartate across both LC–MS/MS and GC–MS platforms (Figures 4 and 5). These four amino acids are either synthesized directly from the glutamine/glutamate reaction (glutamate) or depend on glutamate‐dependent reactions (proline, aspartate, alanine). Figure 4B,D illustrates the metabolic interconnection between glutamine, glutamate, proline, aspartate, alanine and associated roles for TCA cycle metabolite production. Despite this, glutamine abundance remained unchanged, suggesting that hypercapnia may alter either (1) glutamine transport into the mitochondria, potentially via downregulation or dysfunction of the SLC1A5 variant or (2) the activity of GLS, the enzyme responsible for converting glutamine to glutamate. Together, it is evident that elevated CO_2_ exposure induces pronounced shifts in monocytic glutamine metabolism, which may in turn alter the downstream synthesis of proline and aspartate, potentially affecting key mitochondrial‐dependent amino acid biosynthetic pathways. The functional consequences of these alterations remain to be determined. By tracking the incorporation of U‐[^13^C]‐labeled glutamine into downstream metabolites, we aimed to elucidate how hypercapnia influences glutamine utilization and nutrient fate. To our knowledge, this is the first study to employ metabolic tracing approaches to study the effects of hypercapnia. Total intracellular glutamine abundance as well as quantified U‐[^13^C]glutamine enrichment measured by both LC–MS/MS and GC–MS analyses were not significantly altered under hypercapnic conditions. Likewise, no change in U‐[^13^C]glutamine uptake was observed (as per MID distributions), indicating that altered glutamine availability is not a driver of this metabolic adaptation (Figures 4C and 6B); that is, the cells have access to sufficient glutamine availability but utilize it differently in hypercapnia. This is supported by results demonstrating decreased enrichment of the M + 5 isotopologue of glutamate (Figure 6C), indicating altered glutaminolysis at the first mitochondrial conversion step. Furthermore, under hypercapnic conditions, enrichment of the M + 4 isotopologue of aspartate is also significantly reduced (Figure 7B). Notably, the appearance of the M + 4 isotopologue of aspartate indicates that oxaloacetate itself was fully labeled, as it serves as the carbon backbone and primary amine acceptor during the conversion reaction of aspartate transaminase (Figure 7A). Additionally, a modest elevation in total ^13^C enrichment of succinate was observed (Figure 7C), indicative of altered TCA cycle turnover from glutamine‐derived intermediates. Taken together, these U‐[^13^C]glutamine tracing experiments demonstrate selective hypercapnia‐dependent mitochondrial adaptation. Importantly, glutamine is not the sole fuel supporting cellular metabolism, and carbon metabolism is fueled by a complex interplay of multiple nutrients including glucose. While we did observe changes in glucose utilization in hypercapnia, notably a decrease in M + 3 Glyc3P and M + 3 alanine, the effects were more modest for TCA‐cycle intermediates α‐KG, succinate, and malate (all *p* < 0.06) (Figures S12 and S13). Thus, we propose that while glucose utilization is modified in hypercapnia, the effects are less prominent than for glutamine utilization and/or may require additional metabolic stressors for hypercapnia to elicit more significant effects.

While nutrient fate and TCA cycle adaptation are clearly a feature in our models, the underpinning mechanisms of these changes remain to be defined. The biosynthesis of proline, a nonessential amino acid, is essential for immune cell function and stress responses. The enzyme PYCR1 catalyzes the final step of proline synthesis, P5C to proline (Figure 8A). Previous work by Phelan et al. demonstrated that hypercapnia significantly increased *PYCR1 mRNA* levels but decreased proline abundance after just 4 h [12]. This may reflect a compensatory response to decreased proline levels, as cells try to adapt to the CO_2_‐dependent mitochondrial/metabolic changes. Ultimately, like hypercapnia‐dependent increases in mitochondrial DNA upregulation, the adaptation is incomplete, with a clear decrease in PYCR1 protein expression and a marked decrease in proline levels in hypercapnia in both our LC–MS/MS and GC–MS experiments. We also observed a significant reduction in U‐[^13^C]glutamine incorporation into M + 5 proline. Focusing next on the glutamine–proline biosynthetic axis, we observed an upregulation of GLS1 protein expression, accompanied by reductions in glutamate abundance and decreased U‐[^13^C]glutamine enrichment of glutamate (Figure 8), suggesting altered activity through this pathway. Importantly, we want to reiterate that intracellular glutamine abundance remained unchanged, indicating that hypercapnia does not induce states of glutamine deprivation in this model. Our current interpretation is based specifically on the observation that M5‐labeled glutamine directly contributes to M5‐labeled glutamate, a well‐established biochemical relationship between these metabolites. With this said, we cannot fully exclude the possibility that glutamine is simultaneously diverted or preferentially utilized in other metabolic pathways. Moreover, these limitations reflect the inherent challenges of interpreting metabolite pool distribution and isotopic enrichment from biochemical analyses, as these measurements do not fully capture metabolically distinct pools on a subcellular basis.

The increase in mitochondrial GLS1 protein expression despite reduced glutamine‐to‐glutamate conversion warrants further consideration. GLS1 expression is regulated by nutrient availability, transcriptional mechanisms, and cellular metabolic state. For example, increased GLS expression has been reported in starvation and diabetic models, where enhanced amino acid catabolism increases the demand for glutamine metabolism [27]. Similarly, the metabolic profile observed under hypercapnic conditions is suggestive of a catabolic phenotype, consistent with previous studies demonstrating hypercapnia‐induced metabolic adaptations and increased lipid utilization, which is abated when catabolic pathways are inhibited [12, 13, 14, 17, 28]. Therefore, increased GLS1 expression may represent a compensatory response to maintain intracellular glutamate availability. However, it remains unclear whether increased GLS1 expression per mitochondrion reflects a net increase at the whole‐cell level, particularly if mitochondrial abundance is reduced under hypercapnic conditions. Furthermore, the effects of prolonged hypercapnic exposure (e.g., 72 h) on GLS1 expression and glutamine metabolism remain to be determined. Furthermore, GLS enzymatic activity was not assessed in this study. As GLS activity is also regulated post‐translationally and allosterically, increased protein abundance cannot be assumed to reflect increased catalytic activity under hypercapnic conditions.

From a clinical perspective, chronic hypercapnia may have important clinical implications, as reduced proline availability alone (not considering other observed adaptations) could impair key processes such as protein synthesis, collagen synthesis [29], antioxidant defense [30], and immune function [31]. Notably, each of these processes has been described to be altered in hypercapnia in a diverse range of tissues and models [16, 17, 32, 33, 34, 35, 36]. Proline metabolism is also implicated in the development of “trained” macrophages associated with allergic asthma [31], while excessive synthesis of proline via PYCR1 contributes to airway inflammation [37]. Beyond these core metabolic changes, we observe a series of regulatory nodes that are not mutually exclusive, collectively reflecting a complex and interconnected metabolic rewiring.

Given that hypercapnia appears to induce a distinct metabolic phenotype, accompanied by clear adaptation of bioenergetic status, the TCA cycle, and glutaminolysis—suggested by altered nutrient enrichment, metabolite abundance, and molecular protein expression—we hypothesize that the glutamine–glutamate–proline axis specifically alters GLS1 and PYCR1 expression alongside reduced glutamate and proline synthesis. More specifically, we consider the evidence for altered proline metabolism to be most consistent with a direct regulatory effect of hypercapnia. This interpretation is supported by our previous findings demonstrating CO_2_‐dependent changes in *mRNA* transcription and transcript abundance at 4 h [12], followed by reductions in PYCR1 protein expression and proline abundance after 24 h. Collectively, these temporal observations support a model in which hypercapnia‐induced transcriptional regulation contributes to subsequent alterations in protein expression and metabolite abundance. This may represent a key mechanistic node underlying these effects, although it is likely to be only one of several contributing pathways. Moreover, and more importantly, we propose that CO_2_‐driven mitochondrial adaptation may represent a hierarchical upstream event, underpinning these metabolic adaptations and ultimately driving the impairment of overall mitochondrial function in hypercapnia.

### 4.1 Limitations

These are in vitro experiments in THP‐1 monocytes exposed to buffered hypercapnia; thus, the impact of elevated CO_2_ in vivo will be more complex and influenced by additional factors. Notwithstanding, the use of a homogeneous cell line enables a systematic and detailed interrogation of complex metabolic changes compared to primary donor cells or tissues. This is particularly important when performing metabolomic and tracing analyses, which are highly sensitive techniques. We have previously validated key THP‐1 hypercapnia‐dependent responses in primary BMDMs, supporting the appropriateness and physiological relevance of the model. Furthermore, we have previously conducted an in‐depth transcriptional analysis in this model, which has informed this study [12, 15].

Hypercapnia in this experiment is achieved by exposing cells to buffered media equilibrated to 10% CO_2_. This represents a CO_2_ tension that has been observed in patients with an exacerbation of COPD. While this is a relatively high level of CO_2_ to be experienced systemically, it is highly likely that local environmental niches experience this level of CO_2_ or higher. Our use of 10% CO_2_ is lower than concentrations used in several in vitro studies (using 15% or 20% CO_2_) [11, 13, 14, 17], and the buffering of our media allows for determination of CO_2_‐specific effects as opposed to pH effects. Our experimental approach minimizes data fragmentation that currently exists in the field and allows us to establish a foundational framework for what we propose to be a mitochondrial degenerative phenotype in response to pathological CO_2_ exposure.

This study has revealed a range of metabolic changes in response to hypercapnia and comprehensively revealed a CO_2_‐sensitive node in amino acid metabolism at the level of glutamine conversion to glutamate. The precise CO_2_‐dependent mechanisms through which this node of sensitivity is targeted in hypercapnia remain unclear and are beyond the scope of this study. Given the widespread metabolic adaptation with respect to acylcarnitines, phosphatidylcholines, sphingomyelins, and amino acids, it will likely be very difficult to define a single responsible mechanism (if indeed this is the case) given the spectrum of changes occurring at the level of the mitochondria in hypercapnic cells.

## 5 Conclusion

Cell metabolism is highly dynamic. It enables rapid adaptation to environmental cues that influence cell fate and effector functions. Our findings demonstrate that hypercapnia promotes significant metabolic reprogramming in monocytes. Given the role of monocyte infiltration into tissue, as well as hypercapnia in COPD, our data offer novel insights into the metabolic landscape of hypercapnia and cell metabolism. Herein, we provide a comprehensive characterization of the metabolic phenotype of immune cells under hypercapnic conditions—a redistribution of glucose metabolism away from canonical glycolytic branched pathways toward the mitochondria, adaptive remodeling of mitochondrial metabolism, and a distinct reprogramming of amino acid and fatty acid oxidation pathways. This phenotype is clearly distinct from the metabolic effects of hypoxia. The concept of distinct metabolic profiles in hypercapnic skeletal muscle versus hypoxic skeletal muscle has recently been described [38].

In this study, we have sought to reconcile a somewhat fragmented literature on hypercapnia and metabolism with a unified and systematic approach in monocytes. Our findings suggest that hypercapnia induces a complex and multifaceted cascade of effects on cellular metabolism and mitochondrial function, which may or may not be attributable to a single, discrete mechanistic pathway. We reveal, for the first time, using a combination of targeted biochemical experiments and unbiased metabolomic analyses, a discrete node of sensitivity at the level of glutamine conversion to glutamate and downstream pathways. Notably, while hypercapnia is often a systemic condition, affecting all cells, tissues, and organs, the specific outcomes on effector function are likely to be cell type‐dependent. For instance, hypothetically, this may manifest as impaired maintenance of mass or strength in skeletal muscle or reduced barrier integrity in endothelial, epithelial, or fibroblast populations. In the present study, however, we specifically focus on characterizing the metabolic phenotype and the mechanistic nodes responsible for such changes within immune cells.

Looking forward, the multifaceted effects of hypercapnia on the mitochondrion raise important questions regarding its overarching influence on cellular physiology. Hypercapnia appears to progressively remodel mitochondrial metabolism through what seems to be a combination of adaptive and maladaptive responses, including altered nutrient utilization, impaired bioenergetic status, changes in protein expression, and transcriptional reprogramming. Most importantly, hypercapnia induces a CO_2_‐driven remodeling of the mitochondrial organelle. In the context of metabolism, such outcomes may have profound consequences for cell function by influencing bioenergetic availability, biosynthetic output, and overall mitochondrial signaling roles, potentially impacting effector responses. Given this, we must go beyond immune cells and consider the implications of other metabolically active cell types, as they may experience similar mitochondrial adaptations under hypercapnic conditions—yet the effector consequence may be evidently different. Future studies are needed to fully decipher the role of these metabolic adaptations and how they induce functional consequences to better guide the development of improved therapeutic strategies for patients.

## Author Contributions

**Eoin P. Cummins:** conceptualization, funding acquisition, writing – original draft, writing – review and editing, project administration, supervision. **Martina Wallace:** resources, writing – review and editing, conceptualization, investigation, methodology. **Xiaofei Yin:** investigation, writing – review and editing, formal analysis, methodology. **Lorraine Brennan:** methodology, conceptualization, writing – review and editing, resources. **David E. Phelan:** conceptualization, writing – review and editing, formal analysis, investigation. **Ben Reddan:** conceptualization, investigation, writing – original draft, writing – review and editing, formal analysis.

## Funding

This work was partly supported by a Science Foundation Ireland (SFI) Career Development award to Eoin Cummins (SFI 15/CDA/3490) which also supported David Phelan and Catarina Mota. Ben Reddan is supported by a physiology research demonstratorship funded by the UCD School of Medicine.

## Conflicts of Interest

The authors declare no conflicts of interest.

## Supporting information


**Figure S1:** Flow cytometric gating strategy used for flow cytometric analysis of BioTracker405 & Nonyl‐Acridine Orange in THP‐1 Monocytes. (A) Initial gating was performed based on side scatter height (SSC‐H) and forward scatter height (FSC‐H) to distinguish the overall cell population from debris. (B) To further refine the population to single, intact cells, FSC‐A versus FSC height (FSC‐H) was used to exclude doublets and cellular aggregates. (C) Following singlet gating, fluorescence in the V450 (BioTracker 405) or FITC (NAO) channel was assessed using appropriate unstained controls to establish background signal thresholds. These gates were then applied to stained samples to identify and quantify specific fluorescence while minimizing false‐positive events and background noise. (D) Representative example of the deployed gating strategy used to distinguish unstained cells (background controls) from positively stained cells, which serve as positive loading controls in all experimental analyses.

**Figure S2:** Hypercapnia does not alter THP‐1 monocyte proliferation, protein concentration, or viability following 24 h exposure. (A) Cell proliferation, (B) protein concentration, and (C) cell viability, assessed by trypan blue exclusion, in THP‐1 monocytes following 24 h exposure to hypercapnia. Data are presented as fold change relative to the vehicle control for proliferation and protein concentration, and as percentage viability for trypan blue analysis. Data represent six independent biological replicates (*N* = 6). (D) ATP assay performed in THP‐1 monocytes assessing cell titration responses to determine the linear range of luminescence detection. Luminescence was measured using a CLARIOstar plate reader, as described in the Methods. Data are representative of three independent experiments and are presented as mean values for each individual n‐number. (E) A PrestoBlue assay was performed on THP‐1 monocytes exposed to 5% or 10% CO_2_ for 24 h ± oligomycin (10 μM). Resorufin fluorescence was measured using a CLARIOstar plate reader according to the manufacturer's instructions. Data are representative of three independent experiments and are presented as mean values for each individual *n*‐number. Statistical analysis was conducted using a paired *t*‐test. Statistical significance is indicated as follows: *p < 0.05. (F) ATP assay data from Figure 2B with reference data points from Figure 1 overlaid to confirm measurements fall within the optimal linear range. Luminescence was recorded using a CLARIOstar plate reader as described in the Methods. Data are presented as mean values for each individual n.

**Figure S3:** Validation of Roxadustat effect to stabilize HIF‐1α. Western blot analysis of HIF‐1α expression in THP‐1 monocytes to validate Roxadustat mechanism of action. Blots are representative of *N* = 3 independent replicates. Lysates were probed using Revert total protein stain and imaged in the 700 nm channel. Blots were incubated with an appropriate primary antibody followed by a fluorescent secondary fluorophore‐conjugated antibody and imaged in the 800‐nm channel on an Li‐COR imaging system. Relative intensities were quantified using Empiria Studio 3.0 Software.

**Figure S4:** Heatmap analysis of significantly altered metabolites when comparing 5% vs. 10% samples. Metabolite distribution following LC–MS/MS analysis of THP‐1 monocytes exposed to 5% or 10% CO_2_ for 24 h. Samples exposed to 5% CO_2_ are shown in pink, 10% CO_2_ in orange. Data are representative of 10 independent experiments (*N* = 10), with all individual biological replicate data points displayed.

**Figure S5:** Heatmap analysis of significantly altered metabolites when comparing 5% vs 10% samples in the presence of LPS. Metabolite distribution following LC–MS/MS analysis of THP‐1 monocytes exposed to 5% or 10% CO_2_ for 24 h + LPS (2.5 μg mL^−1^ for 22 h). Samples exposed to 5% CO_2_ + LPS in pink, and 10% CO_2_ + LPS in orange. Data are representative of 10 (5%) or 8 (10%) independent experiments (*N* = 10, or *N* = 8), with all individual biological replicate data points displayed.

**Figure S6:** Heatmap analysis of significantly altered metabolites when comparing samples under 5% CO_2_ exposed to a vehicle or LPS. Metabolite distribution following LC–MS/MS analysis of THP‐1 monocytes exposed to LPS (2.5 μg mL^−1^ for 22 h). Samples exposed to 5% CO_2_ are shown in pink 5% CO_2_ + LPS in light pink. Data are representative of 10 independent experiments (*N* = 10), with all individual biological replicate data points displayed.

**Figure S7:** Heatmap analysis of significantly altered metabolites when comparing samples under 10% CO_2_ exposed to a vehicle or LPS. Metabolite distribution following LC–MS/MS analysis of THP‐1 monocytes exposed to LPS (2.5 μg mL^−1^ for 22 h). Samples exposed to 10% CO_2_ are shown in orange, and 10% CO_2_ + LPS in light orange. Data are representative of 10 (Veh) or 8 (+LPS) independent experiments (*N* = 10, *N* = 8), with all individual biological replicate data points displayed.

**Figure S8:** LC–MS/MS metabolomic analysis reveals that LPS significantly alters amino acid and lipid species abundance in THP‐1 monocytes. Volcano plot analysis of metabolites in (A) normocapnia and (B) hypercapnia, analyzing the effect of LPS (2.5 μg mL^−1^ for 22 h) following 24 h exposure to hypercapnia on metabolite abundance. The cutoff for significance is *p*_adj_ < 0.05. Significantly increased metabolites are shown in green, and decreased metabolites are shown in red. Venn diagram representing the number of metabolites that are commonly (C) increased and (D) decreased in normocapnia (pink) and hypercapnia (orange) for 24 h. (E) Table representation of amino acid and lipid species identified from the intersection of Venn diagrams, highlighting 24 LPS‐induced metabolites that were significantly upregulated in normocapnia only. Data represent 10 independent experiments (*N* = 10).

**Figure S9:** Metabolite distribution following LC–MS/MS analysis of THP‐1 monocytes exposed to 5% or 10% CO_2_ for 24 h. (A) Amino acids, (B) acylcarnitines, and (C) biogenic amines. Samples exposed to 10% CO_2_ are colored orange, and 10% CO_2_ + LPS are colored light orange. Data are presented as fold change relative to the corresponding 5% CO_2_ control. Data are representative of 10 independent experiments (*N* = 10), with all individual biological replicate data points displayed. Statistically significant changes observed in both basal and LPS‐stimulated states are highlighted using grouped boxes: green indicates increased abundance, whereas red indicates decreased abundance.

**Figure S10:** Metabolite distribution following LC–MS/MS analysis of THP‐1 monocytes exposed to 5% or 10% CO_2_ for 24 h. (A) Lysophosphatidylcholines, (B) sphingomyelin, and (C) phosphatidylcholines. Samples exposed to 10% CO_2_ are colored orange and 10% CO_2_ + LPS are colored light orange. Data are presented as fold change relative to corresponding 5% CO_2_ control. Data are representative of 8–10 independent experiments (*N* = 8–10), with all individual biological replicate data points displayed. Statistically significant changes observed in both basal and LPS‐stimulated states are highlighted using grouped boxes: green indicates increase abundance, whereas red indicates decreased abundance.

**Figure S11:** Total abundance of U‐[^13^C]glutamine and individual isotopologue distribution. Tracing of U‐[^13^C]glutamine into (A) glutamine, (B) glutamate, (C) proline, and (D) aspartate in THP‐1 monocytes exposed to 5% or 10% CO_2_ for 24 h. All analyses were performed using GC–MS. Isotopologues are shown in increasing intensities of orange shading. Data were generated by multiplying isotopologue labeling by metabolite abundance to represent the total abundance of ^13^C‐glutamine‐derived amino acids. This approach accounts for differences in overall amino acid levels between conditions when assessing label incorporation. Data are representative of six independent experiments (*N* = 6), with all individual biological replicate data points displayed.

**Figure S12:** GC–MS analysis reveals that hypercapnia significantly alters U‐[^13^C]glucose enrichment of glycolytic branche metabolites. (A) Schematic diagram depicting the canonical pathway and carbon distribution of U‐[^13^C]glucose enrichment of the glycolytic pathway. Specific nodes of focus are indicated by blue glowing arrows. Quantification of U‐[^13^C]glucose enrichment of (B) Glyc3P, (C) lactate, (D) alanine, and (E) pyruvate following 24 h exposure to hypercapnia. Data analyzed represent either the total U‐[^13^C]glucose enrichment quantification or analysis of individual isotopologues (Y‐axis). Data represent six independent experiments (*N* = 6) and are presented as mean ± standard deviation. Statistical analysis was conducted using a paired *t*‐test. Statistical significance is indicated as follows: **p < 0.01.

**Figure S13:** GC–MS analysis reveals that hypercapnia alters U‐[^13^C]glucose enrichment of TCA cycle metabolites. (A) Schematic diagram depicting the canonical pathway and carbon distribution of U‐[^13^C]glucose in the TCA cycle. Specific nodes of focus are indicated by blue glowing arrows. Quantification of U‐[^13^C]glucose enrichment of (B) alpha‐ketoglutarate (α‐KG), (C) succinate, and (D) malate following 24 h exposure to hypercapnia. Data analyzed represent the U‐[^13^C]glucose enrichment of individual isotopologues (Y‐axis). Data represent six independent experiments (*N* = 6) and are presented as mean ± standard deviation. Statistical analysis was conducted using a paired *t*‐test. Statistical significance is indicated as follows: *p < 0.05, **p < 0.01.

**Figure S14:** Validation of mitochondrial enrichment protocol. Western blot analysis of (A) mt‐TFAM, (B) VDAC, and (C) PYCR1 expression in THP‐1 monocytes (whole cell versus mitochondrial fractions) to validate significant enrichment of mitochondrial proteins following manufacturer's manual instructions (*N* = 3). Mitochondrial fractions were prepared as per manufacturer's protocol. Lysates were probed using Revert total protein stain and imaged in the 700 nm channel. Blots were incubated with an appropriate primary antibody followed by a fluorescent secondary fluorophore‐conjugated antibody and imaged in the 800‐nm channel on an Li‐COR imaging system. Relative intensities were quantified using Empiria Studio 3.0 Software.

**Figure S15:** Chemical structures of compounds used in this study are shown. The displayed structures include all compounds currently available and provided by manufacturers. These include ETC inhibitor, oligomycin; glucose and U‐[^13^C]glucose; L‐glutamine and U‐[^13^C]glutamine. Please note that the chemical structures for BioTracker 405 are not publicly available and were not disclosed by the manufacturers.

**Table S1:** Preparation of buffered media for varying atmospheric CO_2_ exposures. Appropriate buffering for 0.04%, 5%, and 10% CO_2_ conditions was achieved by adjusting sodium bicarbonate concentrations and supplementing with sodium chloride to correct for osmolarity.

**Appendix S1:** imcb70162‐sup‐0017‐AppendixS1.zip.

## Data Availability

Raw data will be made available on publication.

## References

[1] P. Pahal , M. F. Hashmi , and S. Sharma , “Chronic Obstructive Pulmonary Disease Compensatory Measures,” in StatPearls (StatPearls Publishing, 2025). 30247837

[2] R. J. Hill and R. W. Davis , “The pK of Specific Groups of Proteins. I. The Alpha‐Amino Group of the Alpha Chain of Human CO‐Hemoglobin,” Journal of Biological Chemistry 242, no. 9 (1967): 2005–2012. 6022849

[3] D. Rawat , P. Modi , and S. Sharma , “Hypercapnea,” in StatPearls (StatPearls Publishing, 2025). 29763188

[4] R. Kikuchi , Y. Iwai , T. Tsuji , et al., “Hypercapnic Tumor Microenvironment Confers Chemoresistance to Lung Cancer Cells by Reprogramming Mitochondrial Metabolism In Vitro,” Free Radical Biology & Medicine 134 (2019): 200–214. 10.1016/j.freeradbiomed.2019.01.014 30639568

[5] G. T. Ferguson and M. Gilmartin , “CO_2_ Rebreathing During BiPAP Ventilatory Assistance,” American Journal of Respiratory and Critical Care Medicine 151, no. 4 (1995): 1126–1135. 10.1164/ajrccm/151.4.1126 7697242

[6] A. Titus and D. K. Sanghavi , “Extracorporeal Carbon Dioxide Removal,” in StatPearls (StatPearls Publishing, 2025). 32310464

[7] A. M. Mathews , N. G. Wysham , J. Xie , et al., “Hypercapnia in Advanced Chronic Obstructive Pulmonary Disease: A Secondary Analysis of the National Emphysema Treatment Trial,” Chronic Obstructive Pulmonary Diseases 7, no. 4 (2020): 336–345. 10.15326/jcopdf.7.4.2020.0176 PMC7883913 32877962

[8] M. Dreher , P. C. Neuzeret , W. Windisch , et al., “Prevalence of Chronic Hypercapnia in Severe Chronic Obstructive Pulmonary Disease: Data From the HOmeVent Registry,” International Journal of Chronic Obstructive Pulmonary Disease 14 (2019): 2377–2384. 10.2147/COPD.S222803 PMC6805244 31695357

[9] Y. Chung , F. L. Garden , G. B. Marks , and H. Vedam , “Population Prevalence of Hypercapnic Respiratory Failure From Any Cause,” American Journal of Respiratory and Critical Care Medicine 205, no. 8 (2022): 966–967. 10.1164/rccm.202108-1912LE 35085057

[10] S. Vonderbank , N. Gibis , A. Schulz , et al., “Hypercapnia at Hospital Admission as a Predictor of Mortality,” Open Access Emergency Medicine 12 (2020): 173–180. 10.2147/OAEM.S242075 PMC7326210 32617025

[11] C. U. Vohwinkel , E. Lecuona , H. Sun , et al., “Elevated CO(2) Levels Cause Mitochondrial Dysfunction and Impair Cell Proliferation,” Journal of Biological Chemistry 286, no. 43 (2011): 37067–37076. 10.1074/jbc.M111.290056 PMC3199454 21903582

[12] D. E. Phelan , C. Mota , M. J. Strowitzki , et al., “Hypercapnia Alters Mitochondrial Gene Expression and Acylcarnitine Production in Monocytes,” Immunology and Cell Biology 101, no. 6 (2023): 556–577. 10.1111/imcb.12642 PMC10330468 36967673

[13] A. Jaitovich , M. Angulo , E. Lecuona , et al., “High CO_2_ Levels Cause Skeletal Muscle Atrophy via AMP‐Activated Kinase (AMPK), FoxO3a Protein, and Muscle‐Specific Ring Finger Protein 1 (MuRF1),” Journal of Biological Chemistry 290, no. 14 (2015): 9183–9194. 10.1074/jbc.M114.625715 PMC4423704 25691571

[14] E. Ceco , D. Celli , S. Weinberg , et al., “Elevated CO(2) Levels Delay Skeletal Muscle Repair by Increasing Fatty Acid Oxidation,” Frontiers in Physiology 11 (2020): 630910. 10.3389/fphys.2020.630910 PMC7859333 33551852

[15] D. E. Phelan , B. Reddan , M. Shigemura , J. I. Sznajder , D. Crean , and E. P. Cummins , “Orphan Nuclear Receptor Family 4A (NR4A) Members NR4A2 and NR4A3 Selectively Modulate Elements of the Monocyte Response to Buffered Hypercapnia,” International Journal of Molecular Sciences 25, no. 5 (2024): 2852. 10.3390/ijms25052852 PMC10931687 38474099

[16] M. J. Strowitzki , R. Nelson , M. P. Garcia , et al., “Carbon Dioxide Sensing by Immune Cells Occurs Through Carbonic Anhydrase 2‐Dependent Changes in Intracellular pH,” Journal of Immunology 208, no. 10 (2022): 2363–2375. 10.4049/jimmunol.2100665 35477686

[17] S. M. Casalino‐Matsuda , N. Wang , P. T. Ruhoff , et al., “Hypercapnia Alters Expression of Immune Response, Nucleosome Assembly and Lipid Metabolism Genes in Differentiated Human Bronchial Epithelial Cells,” Scientific Reports 8, no. 1 (2018): 13508. 10.1038/s41598-018-32008-x PMC6131151 30202079

[18] J. M. Petit , J. M. Petit , A. Maftah , M. H. Ratinaud , and R. Julien , “10N‐Nonyl Acridine Orange Interacts With Cardiolipin and Allows the Quantification of This Phospholipid in Isolated Mitochondria,” European Journal of Biochemistry 209, no. 1 (1992): 267–273. 10.1111/j.1432-1033.1992.tb17285.x 1396703

[19] C. A. Fernandez , C. D. Rosiers , S. F. Previs , F. David , and H. Brunengraber , “Correction of 13C Mass Isotopomer Distributions for Natural Stable Isotope Abundance,” Journal of Mass Spectrometry 31, no. 3 (1996): 255–262. 10.1002/(SICI)1096-9888(199603)31:3<255::AID-JMS290>3.0.CO;2-3 8799277

[20] X. Yin , O. Prendiville , A. E. McNamara , and L. Brennan , “Targeted Metabolomic Approach to Assess the Reproducibility of Plasma Metabolites Over a Four Month Period in a Free‐Living Population,” Journal of Proteome Research 21, no. 3 (2022): 683–690. 10.1021/acs.jproteome.1c00440 PMC8902803 34978446

[21] A. C. Selfridge , M. A. S. Cavadas , C. C. Scholz , et al., “Hypercapnia Suppresses the HIF‐Dependent Adaptive Response to Hypoxia,” Journal of Biological Chemistry 291, no. 22 (2016): 11800–11808. 10.1074/jbc.M116.713941 PMC4882447 27044749

[22] M. Picard and O. S. Shirihai , “Mitochondrial Signal Transduction,” Cell Metabolism 34, no. 11 (2022): 1620–1653. 10.1016/j.cmet.2022.10.008 PMC9692202 36323233

[23] G. Paradies , V. Paradies , F. M. Ruggiero , and G. Petrosillo , “Role of Cardiolipin in Mitochondrial Function and Dynamics in Health and Disease: Molecular and Pharmacological Aspects,” Cells 8, no. 7 (2019): 728. 10.3390/cells8070728 PMC6678812 31315173

[24] H. S. Hillen , Y. I. Morozov , A. Sarfallah , D. Temiakov , and P. Cramer , “Structural Basis of Mitochondrial Transcription Initiation,” Cell 171, no. 5 (2017): 1072–1081.e10. 10.1016/j.cell.2017.10.036 PMC6590061 29149603

[25] V. Shoshan‐Barmatz , V. de Pinto , M. Zweckstetter , Z. Raviv , N. Keinan , and N. Arbel , “VDAC, a Multi‐Functional Mitochondrial Protein Regulating Cell Life and Death,” Molecular Aspects of Medicine 31, no. 3 (2010): 227–285. 10.1016/j.mam.2010.03.002 20346371

[26] M. Lee , M. Katerelos , K. Gleich , et al., “Phosphorylation of Acetyl‐CoA Carboxylase by AMPK Reduces Renal Fibrosis and Is Essential for the Anti‐Fibrotic Effect of Metformin,” Journal of the American Society of Nephrology 29, no. 9 (2018): 2326–2336. 10.1681/ASN.2018010050 PMC6115654 29976587

[27] B. I. Labow , W. W. Souba , and S. F. Abcouwer , “Mechanisms Governing the Expression of the Enzymes of Glutamine Metabolism–Glutaminase and Glutamine Synthetase,” Journal of Nutrition 131, no. 9 Suppl (2001): 2467S–2474S; discussion 2486S–7S. 10.1093/jn/131.9.2467S 11533295

[28] B. Reddan and E. P. Cummins , “The Regulation of Cell Metabolism by Hypoxia and Hypercapnia,” Journal of Biological Chemistry 301, no. 3 (2025): 108252. 10.1016/j.jbc.2025.108252 PMC11923829 39914740

[29] S. M. Krane , “The Importance of Proline Residues in the Structure, Stability and Susceptibility to Proteolytic Degradation of Collagens,” Amino Acids 35, no. 4 (2008): 703–710. 10.1007/s00726-008-0073-2 18431533

[30] S. Hayat , Q. Hayat , M. N. Alyemeni , A. S. Wani , J. Pichtel , and A. Ahmad , “Role of Proline Under Changing Environments: A Review,” Plant Signaling & Behavior 7, no. 11 (2012): 1456–1466. 10.4161/psb.21949 PMC3548871 22951402

[31] H. Li , L. Ma , W. Li , et al., “Proline Metabolism Reprogramming of Trained Macrophages Induced by Early Respiratory Infection Combined With Allergen Sensitization Contributes to Development of Allergic Asthma in Childhood of Mice,” Frontiers in Immunology 13 (2022): 977235. 10.3389/fimmu.2022.977235 PMC9533174 36211408

[32] E. P. Cummins , K. M. Oliver , C. R. Lenihan , et al., “NF‐kappaB Links CO_2_ Sensing to Innate Immunity and Inflammation in Mammalian Cells,” Journal of Immunology 185, no. 7 (2010): 4439–4445. 10.4049/jimmunol.1000701 20817876

[33] S. M. Casalino‐Matsuda , F. Chen , F. J. Gonzalez‐Gonzalez , et al., “Myeloid Zfhx3 Deficiency Protects Against Hypercapnia‐Induced Suppression of Host Defense Against Influenza A Virus,” JCI Insight 9, no. 4 (2024): e170316. 10.1172/jci.insight.170316 PMC11143927 38227369

[34] J. B. Dean , “Hypercapnia Causes Cellular Oxidation and Nitrosation in Addition to Acidosis: Implications for CO2 Chemoreceptor Function and Dysfunction,” Journal of Applied Physiology 108, no. 6 (2010): 1786–1795. 10.1152/japplphysiol.01337.2009 PMC2886689 20150563

[35] W. C. Yang , C. Y. Song , N. Wang , et al., “Hypercapnic Acidosis Confers Antioxidant and Anti‐Apoptosis Effects Against Ventilator‐Induced Lung Injury,” Laboratory Investigation 93, no. 12 (2013): 1339–1349. 10.1038/labinvest.2013.118 24126891

[36] M. Shigemura , F. L. Nuñez Santana , S. M. Casalino‐Matsuda , et al., “Hypercapnia Promotes Maladaptive Airway and Vascular Remodeling in Mice,” Journal of Clinical Investigation 135, no. 21 (2025): e196928. 10.1172/JCI196928 PMC12578406 40857108

[37] T. Xu , Z. Wu , Q. Yuan , et al., “Proline Is Increased in Allergic Asthma and Promotes Airway Remodeling,” JCI Insight 8, no. 16 (2023): e167395. 10.1172/jci.insight.167395 PMC10543727 37432745

[38] J. Balnis and A. Jaitovich , “Hypoxia and Hypercapnia Elicit Overlapping but Distinct Skeletal Muscle Toxicities,” Journal of Physiology (2026). 10.1113/JP289079 41733125

